# The Broad Spectrum of *TP53* Mutations in CLL: Evidence of Multiclonality and Novel Mutation Hotspots

**DOI:** 10.1155/2023/4880113

**Published:** 2023-05-09

**Authors:** Grégory Lazarian, Bernard Leroy, Floriane Theves, Myriam Hormi, Rémi Letestu, Virginie Eclache, Giulia Tueur, Adam Ameur, Audrey Bidet, Pascale Cornillet-Lefebvre, Frédéric Davi, Eric Delabesse, Marie-Hélène Estienne, Pascaline Etancelin, Olivier Kosmider, Sophy Laibe, Marc Muller, Nathalie Nadal, Dina Naguib, Cédric Pastoret, Stéphanie Poulain, Pierre Sujobert, Lauren Veronese, Samia Imache, Valérie Lefebvre, Florence Cymbalista, Fanny Baran-Marszak, Thierry Soussi, On behalf of the French Innovative Leukemia Organization (FILO)

**Affiliations:** ^1^Service d'Hématologie Biologique, Hopital Avicenne, Hopitaux Universitaire de Paris Seine Saint Denis, Assistance Publique Hopitaux de Paris, Bobigny, France; ^2^Sorbonne Université, Place Jussieu, 75005 Paris, France; ^3^Service d'Hématologie Biologique, CHU Pitié Salpêtrière, Paris 75013, France; ^4^Science for Life Laboratory, Department of Immunology, Genetics and Pathology, Uppsala University, Uppsala, Sweden; ^5^Service d'Hématologie Biologique, CHU Bordeaux-Haut Lévêque, Bordeaux 33000, France; ^6^Service d'Hématologie Biologique, CHU Hôpital Robert Debré, Reims 51100, France; ^7^Laboratoire d'Hématologie, CHU Toulouse, Inserm 1037, CNRS, Université Toulouse III-Paul Sabatier, Centre de Recherches en Cancérologie de Toulouse, Toulouse, France; ^8^Service d'Hématologie Biologique, CHRU Tours, Tours 37044, France; ^9^Laboratoire de Génétique Oncologique, Département de Biopathologie, Centre Henri Becquerel, 76038 Rouen, France; ^10^Service d'Hématologie Biologique, CHU Hôpital Cochin, Paris 75014, France; ^11^XPath Cytogénétique et Biologie Moléculaire, Marseilles 13010, France; ^12^Laboratoire de Génétique Médicale, CHRU Nancy-Hôpitaux de Brabois, Vandoeuvre-Lès-Nancy, 54511, France; ^13^Service de Génétique Chromosomique et Moléculaire, CHU Dijon, Dijon 21000, France; ^14^Service d'Hématologie Biologique, CHU Caen, Caen 14033, France; ^15^Service d'Hématologie Biologique, CHU Rennes, Rennes 35000, France; ^16^Service d'Hématologie Cellulaire, CHRU Lille, Lille 59000, France; ^17^Service d'Hématologie Cellulaire, Hospices Civils de Lyon, Lyon 69002, France; ^18^Service de Cytogénétique Médicale, CHU Clermont-Ferrand, Clermont-Ferrand 63000, France; ^19^Centre de Recherche Saint-Antoine, UMRS_938, “Hematopoietic and Leukemic Development”, Sorbonne Université, 75012 Paris, France; ^20^Department of Immunology, Genetics and Pathology, Science for Life Laboratory, Uppsala University, SE-751 85 Uppsala, Sweden; ^21^French Innovative Leukemia Organization (FILO), France

## Abstract

*TP53* aberrations are a major predictive factor of resistance to chemoimmunotherapy in chronic lymphocytic leukemia (CLL), and an assessment of them before each line of treatment is required for theranostic stratification. Acquisition of subclonal *TP53* abnormalities underlies the evolution of CLL. To better characterize the distribution, combination, and impact of *TP53* variants in CLL, 1,056 *TP53* variants collected from 683 patients included in a multicenter collaborative study in France were analyzed and compared to UMD_CLL, a dataset built from published articles collectively providing 5,173 *TP53* variants detected in 3,808 patients. Our analysis confirmed the presence of several CLL-specific hotspot mutations, including a two-base pair deletion in codon 209 and a missense variant at codon 234, the latter being associated with alkylating treatment. Our analysis also identified a novel CLL-specific variant in the splice acceptor signal of intron 6 leading to the use of a cryptic splice site, similarly utilized by *TP53* to generate p53psi, a naturally truncated p53 isoform localized in the mitochondria. Examination of both UMD_CLL and several recently released large-scale genomic analyses of CLL patients confirmed that this splice variant is highly enriched in this disease when compared to other cancer types. Using a TP53-specific single-nucleotide polymorphism, we also confirmed that copy-neutral loss of heterozygosity is frequent in CLL. This event can lead to misinterpretation of *TP53* status. Unlike other cancers, CLL displayed a high proportion of patients harboring multiple *TP53* variants. Using both in silico analysis and single molecule smart sequencing, we demonstrated the coexistence of distinct subclones harboring mutations on distinct alleles. In summary, our study provides a detailed *TP53* mutational architecture in CLL and gives insights into how treatments may shape the genetic landscape of CLL patients.

## 1. Introduction

Chronic lymphocytic leukemia (CLL) is a highly heterogeneous disease in terms of clinical outcomes and chemoimmunotherapy responses [1]. Despite improvements in care, CLL remains incurable. Even after prolonged responses to therapy, patients will relapse and thus need multiple lines of treatment [2]. Since the first publication of *TP53* alterations in CLL in the early 1990s, numerous teams have confirmed the high variety and prevalence of *TP53* alterations in the pathology. Predominantly, these alterations manifest as a deletion of the gene on the short arm of chromosome 17 (17p13.1) and a missense mutation in the second allele [3, 4]. *TP53* alterations are relatively infrequent in treatment-naïve CLL patients (10%), but their incidence may reach 50% to 60% in those with fludarabine-refractory disease [5, 6]. In a seminal paper published in 2000, Döhner et al. showed that 17p deletion (del(17p)) was associated with markedly decreased survival and that it predicted impaired response to chemoimmunotherapy [7]. Their findings were confirmed in many subsequent studies, making *TP53* status the main predictive marker in CLL for the selection of appropriate treatments [8–10]. The detection of del(17p) and *TP53* mutations has become an integral part of routine diagnostics and should be performed before any administration of treatment. The advent of next-generation sequencing (NGS) has changed the CLL paradigm [11]. First, it has led to a better understanding of the heterogeneous nature of the disease, with the discovery of multiple driver genes associated with its development. Second, concerning *TP53*, it has shed light on new features such as the occurrence of minor clones, which had remained undetectable by conventional Sanger sequencing [6, 12–16]. The clinical value of these minor clones is still under investigation [17]. In a previous study, using a dataset of 336 *TP53*-mutated CLL patients, we uncovered a novel *TP53* mutation hotspot in codon 234 associated with chlorambucil treatment [18].

In the present study, we collected retrospective data on *TP53*-mutated patients from centers affiliated with the French Innovative Leukemia Organization-CLL (FILO) in GBMHM (French Molecular Biology Group in Hematology) laboratories. All centers contributing to the present work had GBMHM or ERIC (European Research Initiative on CLL) quality control certification [19].

## 2. Results

### 2.1. The FILO and UMD_CLL Datasets

**Figure 1:**
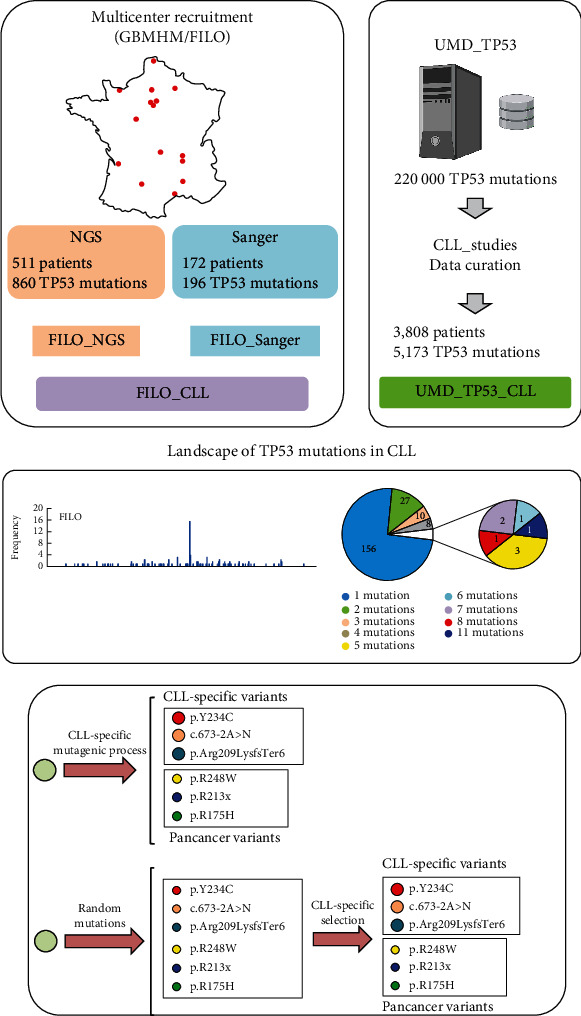
Workflow diagram of the study.

**Figure 2:**
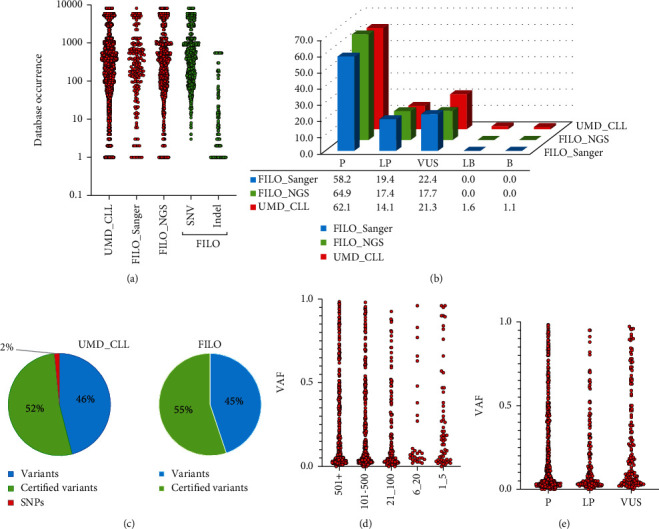
Validation of the FILO dataset. (a) The frequency of *TP53* variants in the FILO dataset is similar to those included in UMD_CLL. The occurrence of each *TP53* variant extracted from UMD_TP53 is shown on the *y*-axis of the graph for three datasets: UMD_CLL: all *TP53* variants from CLL patients included in the UMD_*TP53* database, excluding FILO data (5173 variants); FILO_NGS/FILO_Sanger: data from the subsets of FILO patients analyzed via NGS or Sanger sequencing, respectively. Single-nucleotide variants (SNV) are more frequent than frameshift mutations (Indel). (b) The majority of *TP53* variants in the FILO dataset are either pathogenic (P) or likely pathogenic (LP). *TP53* variants in both datasets were classified according to the pathogenicity data included in the UMD_*TP53* database (B: benign; LB: likely benign; VUS: variant of uncertain significance). (c) More than 50% of *TP53* found in CLL, in both UMD_CLL and the FILO dataset, are certified deleterious *TP53* variants. UMD_CLL includes several benign polymorphisms (SNP) that were removed for all subsequent analyses (Supplementary table S4). (d) VAF is similar depending on the frequency of the *TP53* variants. The number of reported cases for each *TP53* variant is indicated in the *x*-axis. The *y*-axis corresponds to the VAF of each *TP53* variant. (e) VAF is similar among the 3 classes (P, LP, and VUS) of *TP53* variants. The *y*-axis corresponds to the VAF of each *TP53* variant. The Mann–Whitney *U* test showed that there were no statistical differences across the three groups, P, LP, and VUS.

To our knowledge, the FILO dataset is, as of this writing, the largest aggregation of *TP53*-mutated CLL patients. For the present study, it provided 1,056 *TP53* variants collected from 683 patients analyzed either by conventional Sanger analysis (172 patients, 196 *TP53* variants) or by NGS (511 patients, 860 *TP53* variants) (Figure 1 and Supplementary Table S1 to S3). Only variants located in the major transcript (NM_000546) and targeting the main *TP53* isoform (NP_000537) will be discussed here. To extend our comparison and analysis, we used the UMD_CLL dataset. This latter includes all CLL patients from UMD_TP53 (excluding the FILO dataset) and provides 5,173 *TP53* variants (3,808 patients) that have been manually curated to remove duplicate entries (Material and Methods). The FILO dataset includes a mix of variants, extending from rare variants to mutation hotspots, a classical schema seen also in UMD_CLL and other types of cancer (Figure 2(a)). As expected, most of the infrequent or unique variants were associated with insertions or deletions (Indel) (Figure 2(a)). *TP53* variant pathogenicity was defined using the ACMG criteria included in the UMD_TP53 database [20]. Pathogenic (P) or likely pathogenic (LP) variants were identified in 82% of the patients in the FILO dataset. The remaining ones in that collection were labeled as variants of uncertain significance (VUS); no benign or likely benign variants were observed therein (Figure 2(b)). In contrast, 45 patients included in UMD_CLL carried a variant defined initially as benign. However, when verified against the recent release of the new infrequent *TP53* single-nucleotide polymorphism (SNP) datasets, 43 (95.5%) of those variants were redefined as benign polymorphisms (Supplementary Table S4) [21]. These variants were removed from all datasets used for the subsequent analyses. Using a TP53-specific grading system based on the recurrence of *TP53* variants in multiple independent genomic repositories (cancer shared datasets (CSD)), we have previously defined a set of 480 variants as certified oncogenic variants (see Material and Methods) [20]. In the FILO dataset and UMD_CLL, 52% and 55% of variants, respectively, were certified as oncogenic (Figure 2(c)).

A 1% variant allele frequency (VAF) cut-off was used for NGS data included in the FILO dataset, but it did not lead to the selection of spurious mutations as the VAF of uncommon *TP53* variants was similar to that of frequent variants (Figure 2(d)) and furthermore similar among the ACMG classes (Figure 2(e)). We note that 315 patients displayed a VAF between 1% and 5% and thus would not have been identified via conventional sequencing. This included 71 patients with single and 244 patients with multiple mutations. The most common *TP53* mutations in the FILO dataset were missense mutations, accounting for 73% of the total number of variants. Frameshift, inframe, nonsense, and splice variants accounted, respectively, for 11%, 2%, 6%, and 8% for the CLL dataset. These percentages were highly similar to those found in UMD_CLL or other cancer types (Supplementary Figure S1). Analysis of the mutational events targeting the *TP53* gene in patients from both the UMD database and the CLL dataset showed that they displayed a high frequency of GC>AT and AT>GC transitions (Supplementary Figure S2A). GC>AT transitions were predominantly associated with hotspot variants localized in CpG dinucleotides and common to all types of cancer. In contrast, the high frequency of AT>GC transitions found in both the FILO dataset and the CLL patients included in UMD_TP53 was not observed in other cancer types (Supplementary Figure S2A). The same pattern was observed when analyzing data from whole genome sequencing in four CLL samples, indicating that this pattern of mutations is indeed characteristic of CLL (Supplementary Figure S2B).

FISH analysis of del(17p) was performed in 208 (61%) of the next-generation sequenced cases. VAF for *TP53* variants was expectedly higher than 50% in some patients with del(17p). Strikingly however, VAF was also greater than 50% (range: 52%–98%) in 11 cases showing no del(17p), suggesting a partial or total replacement of the wild-type *TP53* locus by the mutant allele. To infer the haplotype of these tumors, we studied 11 SNPs of the *TP53* locus covered by NGS (Supplementary Figure S3A). An analysis of eight cases with no del(17p) and a VAF > 50% showed tumor homozygosity for all SNPs. As expected, heterozygous SNPs were identified in the eight informative cases with VAF < 50% and no del(17p) (Supplementary Figure S3B).

### 2.2. TP53 Hotspot Variants in CLL

*TP53* variants in the whole FILO dataset were predominantly distributed in the DNA-binding domain, with classical CpG-related hotspots at codons 175, 248, and 273, a distribution similar to that observed in other cancers, including hematological malignancies (Supplementary Figure S4A). Nevertheless, three CLL-specific mutation hotspots were observed in the FILO dataset and validated in UMD_CLL. The first was located at codon 234 (NP_000537_p.Tyr234His, NM_000546_c.700T>C), which has a very low frequency of mutation in the majority of cancers (Supplementary Figure S4B). Our previous study on 336 patients showed that this nonfunctional *TP53* variant is found mainly in patients treated with chlorambucil (CLB), an alkylating drug that had been widely used to treat CLL patients before the development of individualized therapy [18]. A survey of the literature and data from UMD_CLL also showed an excess of mutations at codon 234 in CLL compared to other cancer types (Supplementary Figure S4B). In the FILO dataset, we also noticed that two CLB-treated patients carried two different substitutions at codon 234 (NP_000537_p.Tyr234Cys and NP_000537_p.Tyr234Ser) on different alleles (Supplementary Figure S5A). Remarkably, this type of event appeared to be particularly rare in any type of cancer in UMD_TP53 (Supplementary Figure S5B): only six of the 225,000 patients included in the whole database carried multiple missense variants at codon 234. Among them are one AML and five CLL patients, with four of these latter having been treated with CLB [22–24] (Supplementary Figure 5B). The absence of this variant in recent collections of CLL patients in which none received CLB also supports the association between this codon and that treatment. Our finding is reminiscent of the association between exposure to certain carcinogens (aflatoxin B1 in hepatocellular carcinoma and benzo(a)pyrene in lung cancer) and *TP53* hotspot variants in codon 249 or 157 [25].

**Figure 3:**
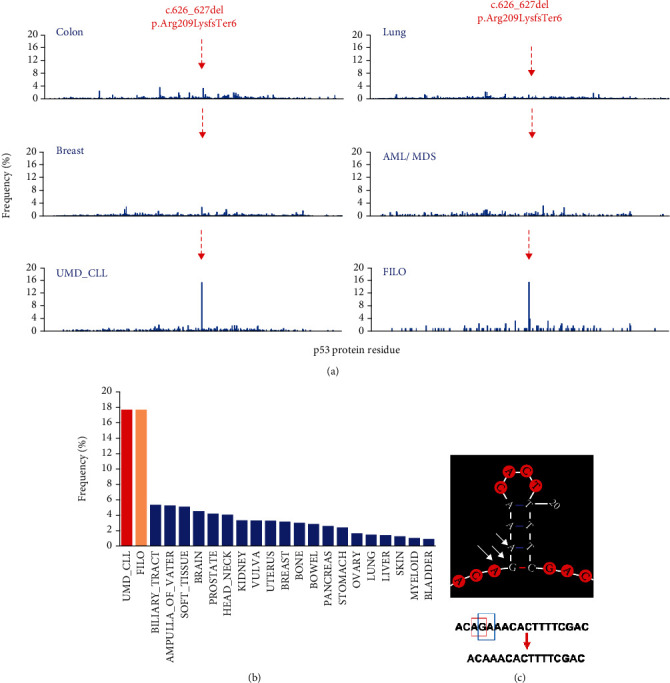
Variant NM_000546_c.626_627del (NP_000537_p.Arg209LysfsTer6) is a hotspot mutation in CLL. (a) Distribution of frameshift mutations at each codon of the *TP53* protein in various types of cancer. Only insertions and deletions are analyzed. The same scale is used for all the analyses to emphasize the CLL hotspot (see Supplementary Figure S5 for more information). AML/MDS: acute myeloid Leukemia and myelodysplastic syndrome. Lung: NSCLC and SCLC. (b) Frequency of NM_000546_c.626_627del (NP_000537_p.Arg209LysfsTer6) in various cancer types included in the UMD_TP53. (c) Potential hairpin structure associated with an inverted repeat in regions 626–628. Two potential mutational events can lead to the same mutation with deletion of either AG or GA depicted by the three arrows. In both the UMD_T53 database and FILO dataset, variant NM_000546_c.626_627del was detected using different methodologies (conventional Sanger sequencing or NGS) precluding any methodological bias.

The overall frequency of frameshift mutations arising from deletions, insertions, or duplications ranges from 5% to 8% among the various types of cancer without obvious hotspot variants as they are scattered along the p53 protein (Figure S1, Figure 3(a), and Supplementary Figure S6). A variant at codon 209 (NM_000546_c.626_627del) leading to premature termination (NP_000537_p.Arg209LysfsTer6) was found to be enriched in CLL (Figure 3(b) and Supplementary Figure S6). The frequency of variant NM_000546_c.626_627del ranged between 1% and 3% for most cancer types but reached 17% in both the FILO dataset and UMD_CLL. This difference was highly significant compared to all other cancer types and defined as a bona fide hotspot for CLL (*p* < 0.0001, chi-square test). This variant had been observed previously in a series of 254 CLL patients [26], and the present analysis showed that it is highly specific to CLL: the concerned region includes an inverted repeat that could explain the high mutability of the sequence *in vivo* (Figure 3(c)). As this variant is observed in multiple reports using different methodologies such as conventional Sanger sequencing or NGS, an association with a methodological bias is unlikely. We also noticed that frameshift mutations at codon 210 in the FILO dataset were also elevated compared to other cancer types (4% versus 0.4% or 0.23% in colorectal and breast cancer, respectively). Whether this high frequency in codons 209 and 210 is due to a paucity of frameshift mutations in other regions of *TP53* in CLL, or to an increase of this event in CLL, is currently unknown, but as discussed below, a CLL-specific selection cannot be excluded.

**Figure 4:**
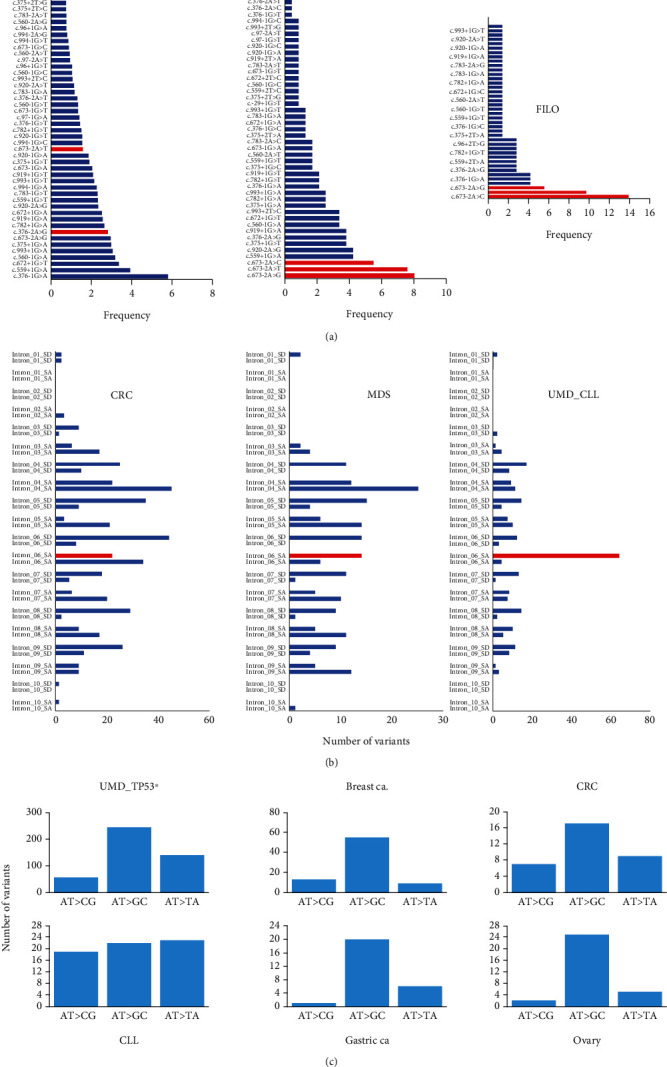
CLL hotspot mutations in intron 6 splice acceptor signal. (a) Frequency of *TP53* mutation in various splice signals included in UMD_TP53 (left panel), UMD_CLL (middle panel) and FILO (right panel). Variants at position NM_000546_c.673-2A are shown in red. (b) Distribution of mutations in the 20 splice signals of the 10 introns of the *TP53* gene. (c) Mutational events at position NM_000546_c.673-2 in various types of cancer. UMD_TP53^∗^: whole *TP53* database without CLL; CRC: colorectal carcinoma; MDS: myelodysplastic syndrome.

**Figure 5:**
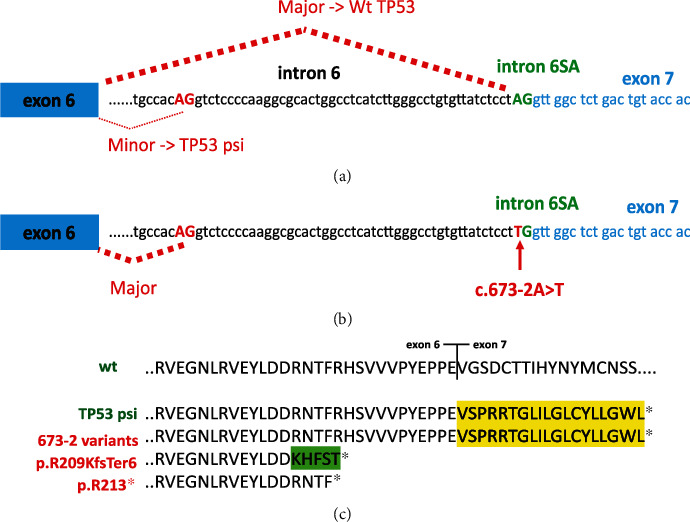
Alternative splicing and mutation consequences in *TP53* intron 6. (a) In unstressed normal cells, full-length wild-type p53 (NP_000537.3, 393 residues) derives from a splice event occurring between exons 6 and 7 (in blue) of the major RNA transcript (NM_000546) using the major splice acceptor site (in green). Upon specific stress, an alternative splice occurs between a cryptic acceptor splice site (in red) localized in the 3′ region of intron 6 leading to the synthesis of a shorter *TP53* isoform (TP53psi). (b) Mutations at position NM_000546_c.673-2 lead to the inactivation of the original acceptor site and the utilization of the cryptic splice acceptor used to generate TP53psi. This consequence has been observed with RNA sequencing analysis in multiple tumors or cell lines bearing variants at position NM_000546_c.672-2A [28, 29]. (c) Putative *TP53* protein variants resulting from various events leading to a truncation of TP53. P53psi or putative variants resulting from a mutation at position NM_000546_c.673-2 bear a new carboxy terminus rising from the translation of intron 6 and finishing with the stop codon in the beginning of exon 7, which is translated in a different reading frame compared to wt p53 (highlighted in yellow). The putative protein, NP_000537_p.Arg209LysfsTer6, expressed by the hotspot variant NM_000546_c.626_627del ends in exon 6 with 5 extra amino acids (highlighted in green). NM_000546_c.637C>T, a hotspot variant found in every type of cancer, truncates *TP53* at codon 213 (NP_000537_p.Arg213Ter).

The third hotspot is located in the splice acceptor signal of intron 6 (Figures 4(a) and 4(b)). Splice mutations (alterations of the canonical ±1 or ±2 splice sites) were underestimated when Sanger sequencing was used to assess *TP53* status. However, more recent exome or whole genome analysis showed that they account for 3% to 6% of *TP53* inactivation in UMD_TP53 with no significant differences among the various histological groups (Supplementary Figure S7A and B). During the analysis of the FILO dataset, we noticed that variants at position NM_000546_c.673-2, the acceptor signal for intron 6, were highly enriched in CLL compared to other cancer types and represented 22% of CLL splice variants (Figures 4(a) and 4(b)). This was observed in the FILO dataset and in UMD_CLL. Catherwood et al. have recently described a collection of 303 untreated *TP53*-mutated patients (429 *TP53* variants) [27]. Unreported by those authors, we noticed that splice variants at position NM_000546_c.673-2A were also the major splice variants. In both UMD_TP53 and UMD_CLL, no other splice site hotspots were identified when analyzing all other cancer types (Supplementary Figure S8). Any one cancer type is characterized by specific patterns of mutational signatures arising from the various mutational processes that have occurred in the tumor (Supplementary figure S2). Analysis of the mutational events at position NM_000546_c.673-2 in various types of cancer showed that the transition A>G is always the major mutational event. In contrast, the three potential substitutions (the transition NM_000546_c.673-2A>T and the two transversions NM_000546_c.673-2A>G and NM_000546_c.673-2A>C) were equally frequent in CLL (Figure 4(c)). It is therefore unlikely that they arise from a single mutational process; rather, these results more likely suggest specific selection for these three splice variants. It should be noted that in CLL, these splice variants in the intron 6 acceptor signal are found at low VAF (range 3% to 15%) predominantly in tumors carrying multiple TP53 variants. RNA-based studies on tumor samples or cell lines bearing these splice variants have shown that a cryptic acceptor splice site located 49 base pairs upstream of the canonical splice site is preferentially used (Figure 5) [28, 29]. In normal cells bearing no *TP53* mutation, this cryptic site is used upon specific stress to generate an alternative transcript expressing p53psi, a truncated isoform that localizes in the mitochondria and displays proproliferative activities despite being unable to bind to DNA and transactivate canonical *TP53* target genes (Figure 5) [30]. Although the expression of this alternative splice variant is inducible upon specific signals, the splice mutation leads to constitutive expression with a potential oncogenic effect. This particular activity of p53psi has also been observed for two other *TP53* variants, i.e., NP_000537_p.Arg196Ter and NP_000537_p.Arg213Ter, the most frequent nonsense variants observed in CLL. That observation suggests that variants truncated in this region are associated with this gain of function [31]. We note that the putative protein NP_000537_p.Arg209LysfsTer6 expressed by hotspot variant NM_000546_c.626_627del may display the same property (Figure 5). Therefore, it is tempting to explain the second and third CLL hotspot mutations described in this section by the specific selection of truncated *TP53* variants with a gain of function specific to CLL.

### 2.3. CLL Patients Harbor Multiple Subclones with Different *TP53* Mutations

**Figure 6:**
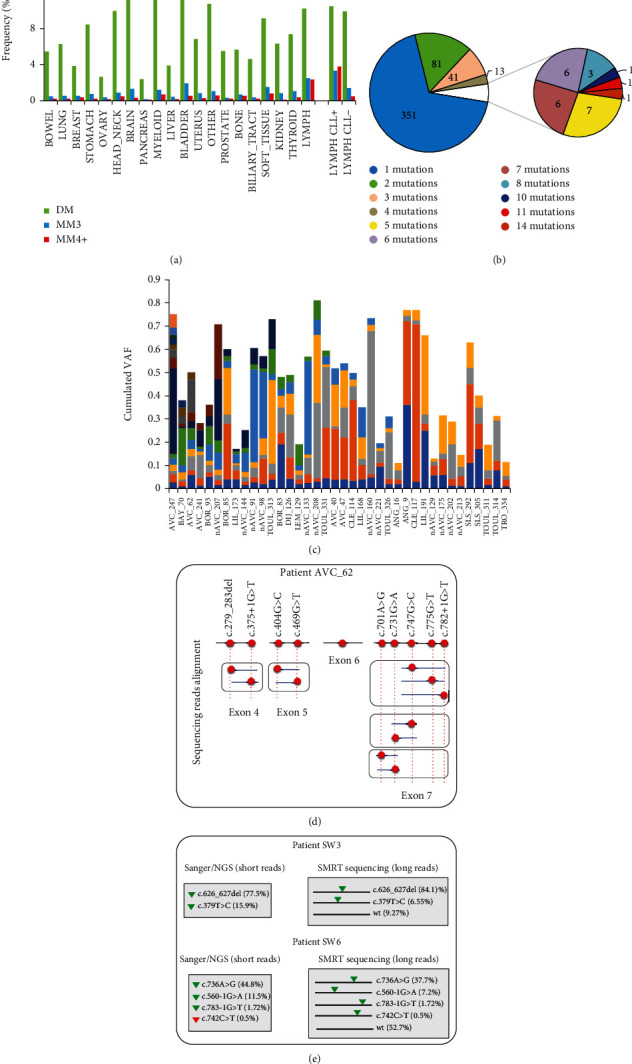
CLL patients are polymutated. (a) *TP53* cancer types classified according to OncoTree were analyzed for tumors carrying two (DM), three (MM3), or more than three (MM4+) *TP53* variants. Lymphoid tumors were split into two subgroups including (CLL+) or excluding (CLL-) CLL patients. (b) Distribution of the number of mutations in tumors from the NGS subset of the FILO dataset. (c) Cumulated VAF from polymutated patients from the NGS subset of the FILO dataset. (d) *TP53* variants are distributed on different chromosomes in the tumor of patient AVC-62. Manual examination of the sequence alignment of NGS data was performed for each exon. For 3 pairs of variants and 1 triplet, *TP53* variants are in a trans configuration. (e) SMRT sequencing shows that *TP53* mutations are on different alleles for patients SW3 and SW6. Standard NGS analysis is shown on the left. No allelic distribution can be inferred from this type of analysis. SMRT sequencing (right) provides an accurate picture of the allelic distribution of each *TP53* variant, as well as the remaining wt allele. The frequencies of the different alleles are shown in brackets. Green triangle: *TP53* variants identified by both types of analyses. Red triangle: *TP53* variants not detected by long-range sequencing (see also Supplementary Figure S10a to S10e for more patients analyzed by SMRT sequencing).

The presence of multiple *TP53* mutations in tumors is, generally speaking, uncommon. However, an analysis of the most recent issue of UMD_TP53 showed that multiple mutations were far more frequent in CLL, with a wide range of mutations per patient (Figure 6 and Supplementary figure S9A-D). Previous studies using functional analysis of separated alleles in yeast (FASAY), a TP53-specific functional assay, had already identified patients carrying different *TP53* variants and the use of NGS has further expanded this observation. In the NGS subset of the FILO dataset, 160 patients (31%) were shown to express multiple *TP53* variants with 81, 41, and 38 patients showing two, three, or more than three variants per tumor (range 2 to 14) (Figure 6(b)). The same trend was observed in independent reports included in UMD_CLL with *TP53* mutations ranging from 2 to 37 per patient (Supplementary figure S9B-D). It is generally assumed that this high burden of *TP53* mutations is associated with the important clonal heterogeneity of CLL. However, that assumption has never been fully investigated.

**Figure 7:**
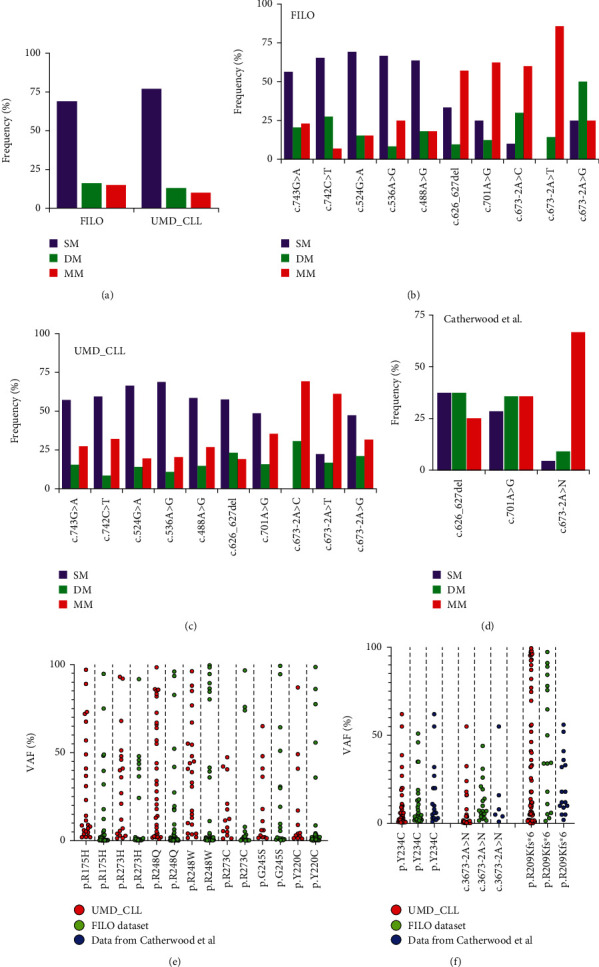
CLL-specific *TP53* variants are observed predominantly in low VAF polymutated patients. (a) Frequency of patients with one (SM), two (DM), or more than two (MM) *TP53* mutations per tumor. (b–d) Frequency of individual *TP53* variants in tumors bearing one (SM), two (DM), or more than two (MM) *TP53* variants in the FILO dataset (b), UMD_CLL (c), or the dataset of Catherwood et al. (e, f) VAF distribution for classical *TP53* hotspot variants (e) or CLL-specific variants (f) in different datasets.

For patients from the FILO dataset analyzed via NGS and expressing multiple *TP53* variants, the cumulated VAF of those variants never exceeded 100%, suggesting that most of them were on different alleles or in independent subclones (Figure 6(c)). This was confirmed by the analysis of individual DNA sequencing reads harboring mutations within the same exon and no more than 50 nucleotides apart, as all *TP53* variants were always in a trans configuration confirming their status as driver mutations (passenger mutations would have been distributed randomly in cis and trans configurations) (Figure 6(e) and Supplementary Figure S10A to S10P). Patient AVC_62, with del(17p) and 10 different *TP53* variants, was particularly informative as several of the mutations were in close proximity with no cis configuration (Figure 6(e)). Five CLL samples from another institution (Karolinska Hospital) were also analyzed using a third-generation, single-molecule real-time (SMRT) sequencing platform (RS II instrument, PacBio, Menlo Park, California) offering long read lengths able to span the most-frequently mutated region of the *TP53* gene [32] These samples, which had been previously tested by NGS and shown to harbor multiple *TP53* variants, also showed only trans configuration (Figure 6(f) and supplementary Figure S11A to S11E). Tumors carrying two *TP53* variants may result from biallelic mutations. However, because we observed a similar frequency of these multimutated tumors for cases with or without del(17p) (Supplementary Figure S12), it appears more likely that such mutations are associated with multiple subclones, but we cannot formally exclude a cis configuration in a single subclone. Although nonfunctional *TP53* variants are associated with CLL, it is well established that there is also an important heterogeneity among the various *TP53* variants with either a simple loss of function, a dominant negative activity, or, for some variants, a gain of function that can vary among cancer types. Multiple classical hotspot variants at codons 175, 248, or 273 can be observed in the same patient, indicating that these alterations are unrelated and ruling out the possibility of any associated bystander effect that could drive the selection/expansion of weaker variants (Supplementary Figure S13). The frequency of these classical hotspot variants as single alterations ranged from 50% to 70%, which accords with the frequency of tumors expressing a unique *TP53* variant (Figures 7(a)–7(c)). Furthermore, their VAF distribution ranges widely from 1% to values greater than 95% (Figure 7(e)). In contrast, hotspot splice variants at position 673-2A are found predominantly in *TP53* polymutated patients with VAFs never exceeding 50% (Figures 7(b)–7(f)). This observation holds true for FILO, UMD_CLL, and data from Catherwood et al.

## 3. Discussion

*TP53* mutation in CLL is a paradigm for translational research. First, it is among the few cancers where *TP53* alterations have been incorporated into routine clinical diagnostics to improve patient stratification, and, second, the landscape of *TP53* mutations in CLL finds no equivalent in other cancer types and inspires research to improve our understanding of this still incurable disease [10, 33–35]. We present here an analysis of the largest set of *TP53* mutations in CLL patients to date with 1,056 *TP53* variants from 683 patients recruited within the centers of the French Innovative Leukemia Organization-CLL (FILO) and 5,173 variants from UMD_CLL, a carefully curated database of *TP53* mutations.

As compared to all other cancer types, our analysis highlights the highly specific landscape of *TP53* alterations in CLL, one comprising three distinct features: (i) a high prevalence of *TP53*-mutated minor clones (VAF below 5%), (ii) an important intratumoral heterogeneity with multiple subclones expressing different *TP53* variants, and (iii) multiple CLL-specific mutation hotspots.

FASAY and thereafter NGS have shown that CLL patients often harbor minor clones expressing pathogenic *TP53* variants (VAF 1%–5%) that progress during the course of disease [12, 22, 36, 37]. There are still some controversial issues regarding the limit of detection (LOD) used for reporting *TP53* variants. The latest recommendations from the *TP53* Network of the European Research Initiative on Chronic Lymphocytic Leukemia (ERIC) advocate for the use of 10% VAF [10]. However, multiple studies have reported variants at lower VAFs [12, 14, 38]. In the present study, 14% of the patients analyzed via NGS harbored a single *TP53* variant with a VAF between 1% and 5% that would have been missed by conventional Sanger sequencing (LOD = 10%–15%). In the era of targeted therapies, chemoimmunotherapy is not recommended for CLL cases with *TP53* mutation or 17p deletion [39]. Thus, the determination of a cut-off able to identify patients with mutated or unmutated *TP53* has become important for therapeutic choices. In their recent laboratory practice recommendations, the GBMHM (*Groupe des biologistes moleculaires des hémopathies malignes*) established rules and quality control standards for the validation of a clinically applicable cut-off value (between 1% and 2%) [19]. The present work shows that this value did not lead to the inclusion of spurious variants with no impact on *TP53* activity, as variants expressed by the above-mentioned minor clones are similar to those found in larger clones with analogous hotspot variant distribution. Moreover, considering the deleterious effect of chemoimmunotherapy in *TP53*-mutated cases and the availability of Btk inhibitors, it seems adequate to consider patients harboring small *TP53*-mutated variants as candidates for targeted therapies [17].

In the setting of our study, most of the *TP53* mutations targeted the DNA-binding domain of the protein and led to *TP53* loss of function in a fashion similar to other cancer types. However, we did uncover some CLL-specific particularities observed both in the FILO dataset and the CLL data in the UMD database. For example, a high, 20% frequency of AT>GC transition in *TP53* was observed in both datasets. That specific transition is seen at a frequency of only 5% to 10% in solid tumors. Similar observations of frequencies of AT>GC transition between 20% and 25% distributed over the entire genome have been made using whole genome sequencing in four untreated CLL patients as well as in 11 patients with monoclonal B-cell lymphocytosis and five patients with ultrastable CLL (>10 years without progression from initial diagnosis) [40, 41]. The etiology of this particular signature is currently unknown, but its detection in untreated patients indicates that it may be a specific feature of CLL.

In this study, using specific *TP53* SNPs, we identified eight patients without del(17p) but with copy-neutral (CN) LOH (also known as uniparental disomy (UDP)). However, our numbers likely underestimate the frequency of CN-LOH in CLL. Because of the presence of multiple clones with different *TP53* variants at low frequency, we cannot exclude CN-LOH in minor clones, and only single-cell sequencing can provide a more accurate characterization of the landscape of this particular genetic event. The question as to whether clones with *TP53* mutations and CN-LOH have the same clinical value as those with a single mutation with or without del(17p) remains unanswered. Also of importance is that *TP53* variants associated with CN-LOH can easily be mistakenly considered as clones with a single *TP53* variant without LOH. This may blur several types of analyses such as the definition of dominant-negative activity of mutant *TP53* toward the wild-type protein.

Three CLL-specific variants were highlighted by the present analysis. First, we discovered that variants in the splice acceptor signal position NM_000546_c.673-2 are specifically enriched in CLL compared to all other cancer types and found predominantly in patients carrying multiple *TP53* variants, a specific feature observed both in the FILO dataset and in UMD_CLL. Although alterations in splice donor sites are predicted to be deleterious, RNA sequencing data analysis from different types of tumors or cell lines has shown that these particular *TP53* mutations lead to the use of an intronic cryptic splice site, with a partial intron retention and the synthesis of a specific protein isoform, p53psi [28, 30]. Upon specific stress, p53psi, lacking a nuclear localization signal, is translocated to the mitochondria and interacts with cyclophilin-D, which leads to an increase in mitochondrial pore permeability and reactive oxygen production [30]. Whether or not TP53psi is expressed in CLL patients and leads to a specific phenotype is currently unknown. However, the high specificity of this variant in CLL indicates that it may merit investigation. Aberrant splicing is a common feature in CLL, and recurrent mutations in SF3B1 can be found in 5% of patients at presentation and climb to 20% during disease progression. These mutations are predominantly subclonal events and associated with more aggressive disease and shorter survival. All these features are similar to those observed for *TP53*, but alterations in these two genes have been shown to be exclusive. Deep analysis of splicing events associated with SF3B1 mutations shows that they induce the use of cryptic 3′ AG signal splices leading to aberrant splicing and partial retention of the 3′ intronic region, which is similar to the outcome of the *TP53* splice hotspot variant at position c.673-2A [42]. *TP53* was not shown to be among the aberrantly spliced mRNA in SF3B1-mutated patients, but it could be mirrored by these specific hotspot mutations.

Individual *TP53* frameshift mutations are, generally speaking, rare. With a frequency ranging between 1% and 5% of all frameshift mutations, variant NM_000546_c.626_627del (NP_000537_p.Arg209LysfsTer6) is nonetheless one of the most frequent of them. Here, in the setting of CLL, we found that the frequency of this variant reached 16% (both in the FILO and the UMD datasets) and that it was the main hotspot for frameshift mutation. The frequency of NM_000546_c.626_627del in CLL had already been brought to light in two independent studies [26, 43]. Our results thus strengthen that observation and clearly define this variant as a CLL-specific hotspot. The coding region residues 625–630 contain an inverted repeat sequence of four nucleotides separated by a four-base spacer, a structure known to cause insertions and/or deletions during replication [44]. Whether or not this particular event is linked to a specific genetic defect in CLL remains to be determined. It should be noted that the putative protein expressed by this frameshift variant is highly similar to p53psi or variant NP_000537_p.Arg213Ter, suggesting that all these variants resulting from different mutational events could be selected for a yet to be discovered gain of function in CLL. The third CLL-specific variant is located at codon 234 with a high prevalence of tyrosine-to-cysteine substitution. This variant, found at very low frequency in all tumor types, has been shown to be specifically associated to CLB treatment [18]. It remains to be determined whether this variant results directly from a mutational event provoked by CLB or is specifically selected during the treatment.

The present study also shed light on the important clonal heterogeneity of CLL, with 113 cases (33%) presenting multiple pathogenic *TP53* variants (up to 11 in a single patient). In a previous study, we showed that tumors with multiple *TP53* mutations were more frequent in lymphoma and leukemia than they were in solid tumors [32]. In that work, we also used SMRT sequencing to demonstrate that *TP53* variants were always distributed in different alleles in acute lymphocytic leukemia (ALL) and myelodysplastic syndrome (MDS), confirming the clonal heterogeneity of those tumors. Using the same methodology here, we confirmed that these mutations were also located in different alleles in all of the analyzed CLL samples. This supplementary observation and the various large-scale analyses of the CLL genome strongly support the multiclonality of these tumors [6, 12–14, 16, 45]. As described in our previous study, the intratumoral genetic heterogeneity in CLL raises the question of the impact of treatment on the selection or acquisition of *TP53* mutations [18]. Longitudinal studies have described the acquisition of *TP53* abnormalities and complex karyotypes in treated relapsed or refractory patients [37]. CLL patients who undergo therapy will ultimately relapse, resulting in the need for multiple lines of therapy with various combinations of drugs. We observed a correlation between the number of *TP53* variants per patient and previous treatments. Any line of treatment significantly increased the number of mutations per patient, but some treatment types did so more than others. Any regimen including the continuous administration of the alkylating agent CLB, whether alone or with other subsequent treatments, dramatically increased the number of mutations per patient [18].

The analysis of *TP53* gene alteration in CLL acts as a magnifying glass on two important features: first, heterogeneity in the setting, with a high number of minor clones, and second, *TP53* addiction with multiple variants selected during disease evolution. Recent studies using single-cell analyses of CLL tumors have identified a high level of genetic and epigenetic heterogeneity beyond *TP53* alteration. Those observations confirm that CLL is not a stable entity, but rather a dynamic disease characterized by a heterogeneous subclonal architecture with a complex course over time shaped by endogenous and exogenous selection pressures [2, 46].

In conclusion, our data here and those of our previous study emphasize the importance of an adequate limit of detection when using NGS for patient stratification [18]. They also highlight the important effects of treatment on clonal heterogeneity and the specific deleterious impact of continuous chlorambucil on both the type and the number of mutations (Box 1). Considering our results, we feel it is necessary to recommend an assessment of the clonal architecture of *TP53* mutations at each line of treatment, in order to limit the use of therapies promoting clonal evolution. In the future, the accumulation of data following long-term targeted therapies is warranted to optimize treatment sequences.

## 4. Material and Methods

### 4.1. Patient Datasets

For the FILO dataset, we collected the retrospective data of *TP53*-mutated patients from centers affiliated with the French Innovative Leukemia Organization-CLL (FILO) in GBMHM (French Molecular Biology Group in Hematology) laboratories (2012 to 2022). All centers contributing to the present work had GBMHM or ERIC (European Research Initiative on CLL) quality control certification [19]. Compared to the NGS datasets used for the previous analysis of codon 234 (336 patients, 568 *TP53* variants), the present dataset included 175 new *TP53*-mutated patients, enabling the identification of a total of 860 *TP53* variants detected in 511 different patients. The whole coding sequence of TP53 including exon-intron junctions was screened by all centers. We also included 172 patients (196 *TP53* variants) analyzed via Sanger sequencing (exons 4 to 8). For NGS analysis, either Illumina or Ion Torrent technologies were used with a VAF of 1% as recommended by the GBMHM. Raw data were aligned using different bioinformatic tools such as GATK or the torrent suite. Minimal average base coverage depth was around 5000x, with a minimal coverage for variant calling at 1000x with at least 10 variant reads and a VAF cut-off of 1%. Target base coverage at 100x was over 98%. Pathogenicity assessment of all variants was performed according to ERIC guidelines [10]. Polymorphisms were carefully excluded using the new *TP53* SNP data included in the most recent version of the UMD_TP53 database [21, 47]. The rate of detected TP53 mutation was around 20%.

### 4.2. The UMD_TP53 Database and In Silico Analysis of *TP53* Variants

The latest issue of the database (2022_R1) includes 207,168 *TP53* variants and data from multiple large-scale tumor analyses such as TCGA GENIE and MSKSCC. This version of UMD_TP53 now includes the OncoTree cancer classification developed by Kundra et al. [48]. It includes nearly 900 tumor types classified into 32 organ sites. Having both tumor types and OncoTree classification in UMD_TP53 increased the specificity of the various analyses. For an accurate analysis, data from all CLL studies were checked manually to remove duplicate entries resulting from the use of the same patients in independent studies. Thus, in all, 4,084 *TP53* somatic variants were extracted and included in UMD_CLL. No specific filtering was applied to remove any variant during this process. FILO data were not included in UMD_CLL for the analyses performed in the present study. For nine studies using NGS (excluding the present analysis), VAF for *TP53* variants was included in the database. The classification of *TP53* variant pathogenicity, in accordance with ACMG criteria and based on population data and *TP53-*specific functional information, has been previously described (https://p53.fr/tp53-database and http://vps338341.ovh.net/) [20].

We recently developed a novel concept to define *TP53* oncogenic driver variants based on their simultaneous occurrence in independent large-scale datasets such as UMD_*TP53* (variants defined by Sanger sequencing only), TCGA, ICGC, and MSKCC Impact [20]. Analysis of all *TP53* variants included in this cancer shared dataset (CSD) using the various large-scale functional assays available confirmed that all these variants have lost their tumor suppressive function. Although the high stringency of this selective procedure does not enable the capture of all oncogenic *TP53* variants, it does lead to the selection of *those* that can be defined nonambiguously as not functional [20].

### 4.3. SMRT Sequencing of TP53 Amplicons

A 2.8 kb amplicon encompassing exons 4 to 8 was used for SMRT analysis. This region includes the majority of the mutations detected in patients and several common *TP53* SNPs useful for phasing the various mutations on the two alleles.

The *TP53* amplicons underwent DNA damage repair and end repair before ligation of hairpin adaptors to generate SMRTbell libraries for circular consensus sequencing. Libraries were then subjected to exotreatment and PB AMPure bead wash procedures for clean-up. Each library was loaded onto one SMRTcell and sequenced on the PacBio RS II instrument using C4 chemistry, P6 polymerase, and a 240-minute movie time. The detection and phasing analyses of *TP53* have been previously described [32].

### 4.4. Statistical Analysis

Statistical analyses were performed with GraphPad Prism 9.0 (GraphPad Software, Inc., San Diego, CA).

## Figures and Tables

**Box 1:**
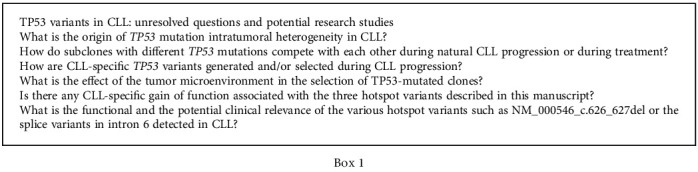
TP53 variants in CLL: unresolved questions and potential research studies.

## Data Availability

TP53 mutations from the FILO cohort used in this study are included within the supplementary information file (Table S3). TP53 mutations included in the UMD database are available from the TP53 web site (https://p53.fr/) or from the corresponding authors upon request.

## Supplementary Materials

Supplementary Materials Supplementary 1: Supplementary Figure S1: genetic landscape of TP53 alterations. Supplementary Figure S2: high frequency of AT>GC transition in CLL. Supplementary Figure S3: copy-neutral LOH (CN-LOH) is frequent in CLL and can be validated by SNP analysis. Supplementary Figure S4: identification of TP53 mutation hotspots in CLL in codon 234. Supplementary Figure S5: multiple TP53 variants in codon 234 in a same patient are associated with CLB exposure. Supplementary Figure S6: variant NM_000546_c.626_627del (NP_000537_p.Arg209LysfsTer6) is a hotspot mutation in CLL. Supplementary Figure S7: frequency of splice mutations in the TP53 gene among the various types of cancer. Supplementary Figure S8: CLL hotspot mutation in the splice acceptor signal of intron 6. Supplementary Figure S9: wide variant distribution in the number of TP53 variants in CLL patients. Supplementary Figure S10 to 10p: TP53 mutations in FILO-cohort polymutated patients are in different alleles. Supplementary figure S11 A to E: long-range sequencing of CLL patients shows that TP53 mutations in multi-mutated patients are in different alleles. Supplementary Figure S12: multiple TP53 variants are found at the same frequency in patients with or without 17p deletion. Supplementary Figure S13: distribution of TP53 hotspot variants in CLL patients. Supplementary Table S1: patient characteristics. Supplementary Table S2: mutations summary. Supplementary Table S4: benign polymorphisms identified in CLL patients included in UMD. Supplementary 2: Supplementary Table S3: mutation description (provided as an Excel file).
