# Supplementary material for: The Broad Spectrum of TP53 Mutations in CLL: Evidence of Multiclonality and Novel Mutation Hotspots
Source: Hum Mutat. 2023 May 9;2023:4880113. doi: 10.1155/2023/4880113 (PMC11918887; doi:10.1155/2023/4880113)
Supplement: Supplementary Materials — Supplementary 1: Supplementary Figure S1: genetic landscape of TP53 alterations. Supplementary Figure S2: high frequency of AT>GC transition in CLL. Supplementary Figure S3: copy-neutral LOH (CN-LOH) is frequent in CLL and can be validated by SNP analysis. Supplementary Figure S4: identification of TP53 mutation hotspots in CLL in codon 234. Supplementary Figure S5: multiple TP53 variants in codon 234 in a same patient are associated with CLB exposure. Supplementary Figure S6: variant NM_000546_c.626_627del (NP_000537_p.Arg209LysfsTer6) is a hotspot mutation in CLL. Supplementary Figure S7: frequency of splice mutations in the TP53 gene among the various types of cancer. Supplementary Figure S8: CLL hotspot mutation in the splice acceptor signal of intron 6. Supplementary Figure S9: wide variant distribution in the number of TP53 variants in CLL patients. Supplementary Figure S10 to 10p: TP53 mutations in FILO-cohort polymutated patients are in different alleles. Supplementary figure S11 A to E: long-range sequencing of CLL patients shows that TP53 mutations in multi-mutated patients are in different alleles. Supplementary Figure S12: multiple TP53 variants are found at the same frequency in patients with or without 17p deletion. Supplementary Figure S13: distribution of TP53 hotspot variants in CLL patients. Supplementary Table S1: patient characteristics. Supplementary Table S2: mutations summary. Supplementary Table S4: benign polymorphisms identified in CLL patients included in UMD. Supplementary 2: Supplementary Table S3: mutation description (provided as an Excel file). [file 4880113.f1.zip › Sup document formated 2023-04-12.pdf]

Title

The broad spectrum of *TP53* mutations in CLL: evidence of multiclonality and novel mutation hotspots.

Running title (60 max)

Spectrum of *TP53* mutations in CLL

Lazarian et al.

Supplementary materials

Supplementary Figure S1: Genetic landscape of *TP53* alterations.

Supplementary Figure S2: High frequency of AT>GC transition in CLL.

Supplementary Figure S3: Copy-neutral LOH (CN-LOH) is frequent in CLL and can be validated by SNP analysis.

Supplementary Figure S4: Identification of *TP53* mutation hotspots in CLL in codon 234.

Supplementary Figure S5: Multiple *TP53* variants in codon 234 in a same patient are associated with CLB exposure.

Supplementary Figure S6: Variant NM_000546_c.626_627del (NP_000537_p.Arg209LysfsTer6) is a hotspot mutation in CLL.

Supplementary Figure S7: Frequency of splice mutations in the *TP53* gene among the various types of cancer.

Supplementary Figure S8: CLL hotspot mutation in the splice acceptor signal of intron 6.

Supplementary Figure S9: Wide variant distribution in the number of *TP53* variants in CLL patients.

Supplementary Figure S10 to 10p: TP53 mutations in FILO-cohort polymutated patients are in different alleles.

Supplementary figure S11 A to E: Long-range sequencing of CLL patients shows that *TP53* mutations in multi-mutated patients are in different alleles.

Supplementary Figure S12: Multiple *TP53* variants are found at the same frequency in patients with or without 17p deletion.

Supplementary Figure S13: Distribution of *TP53* hotspot variants in CLL patients.

Supplementary Table S1: Patient characteristics.

Supplementary Table S2: Mutations summary.

Supplementary Table S3: Mutation description. (Provided as an Excel file)

Supplementary Table S4: Benign polymorphisms identified in CLL patients included in UMD.

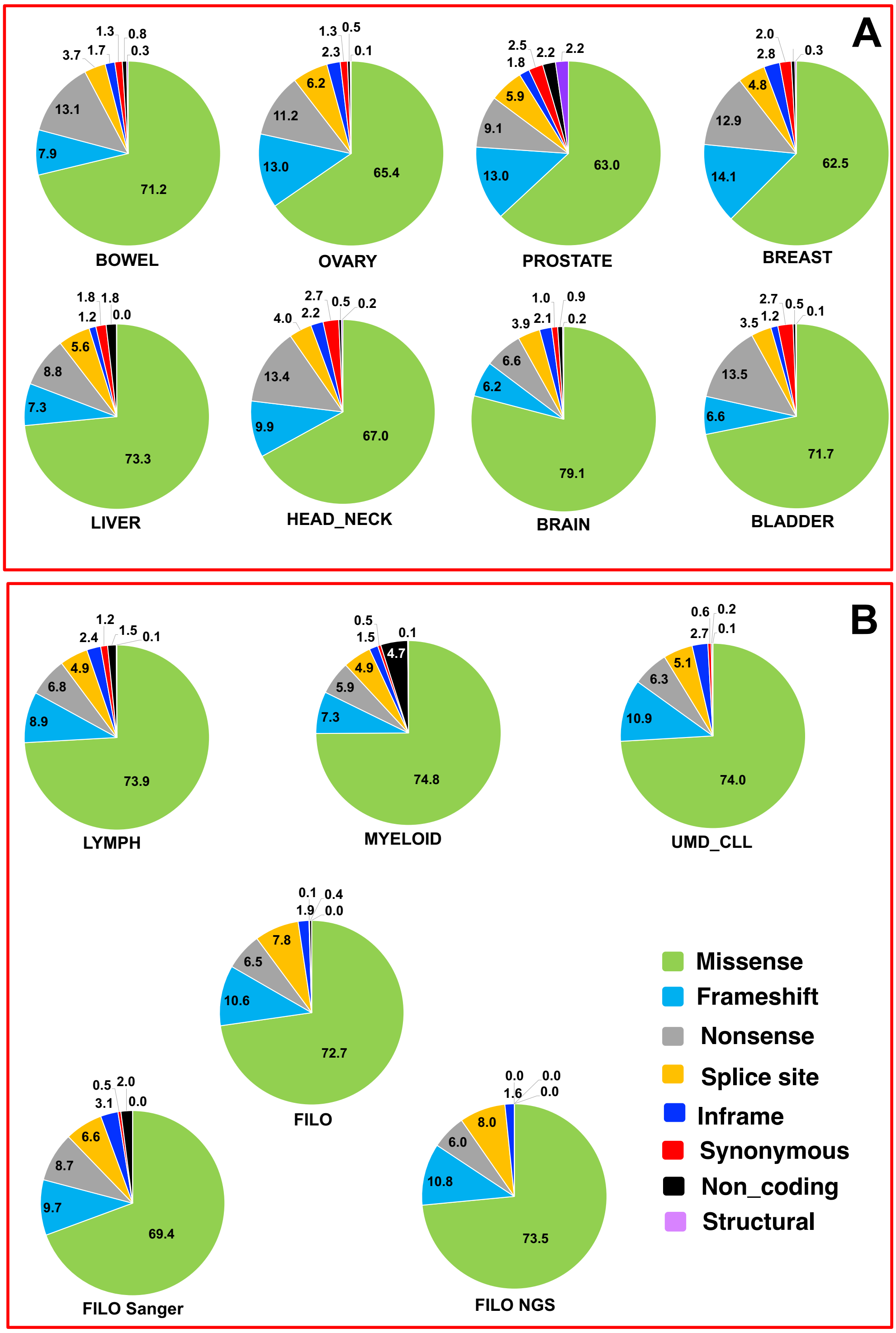

Supplementary Figure S1: Genetic landscape of *TP53* alterations.

A: Data from UMD_TP53 (R2, 2022) for solid tumors. **B**: Data from UMD_TP53 (R2, 2022) for hematological neoplasia. LYMPH and MYELOD: all lymphoid and myeloid neoplasms respectively. UMD_CLL: all chronic lymphocytic leukemia except FILO data. FILO: data from the whole FILO cohort or sequenced via Sanger (FILO Sanger or NGS 'FILO NGS). Organ site definition for all tumors in UMD was performed using the OncoTree classification system [1].

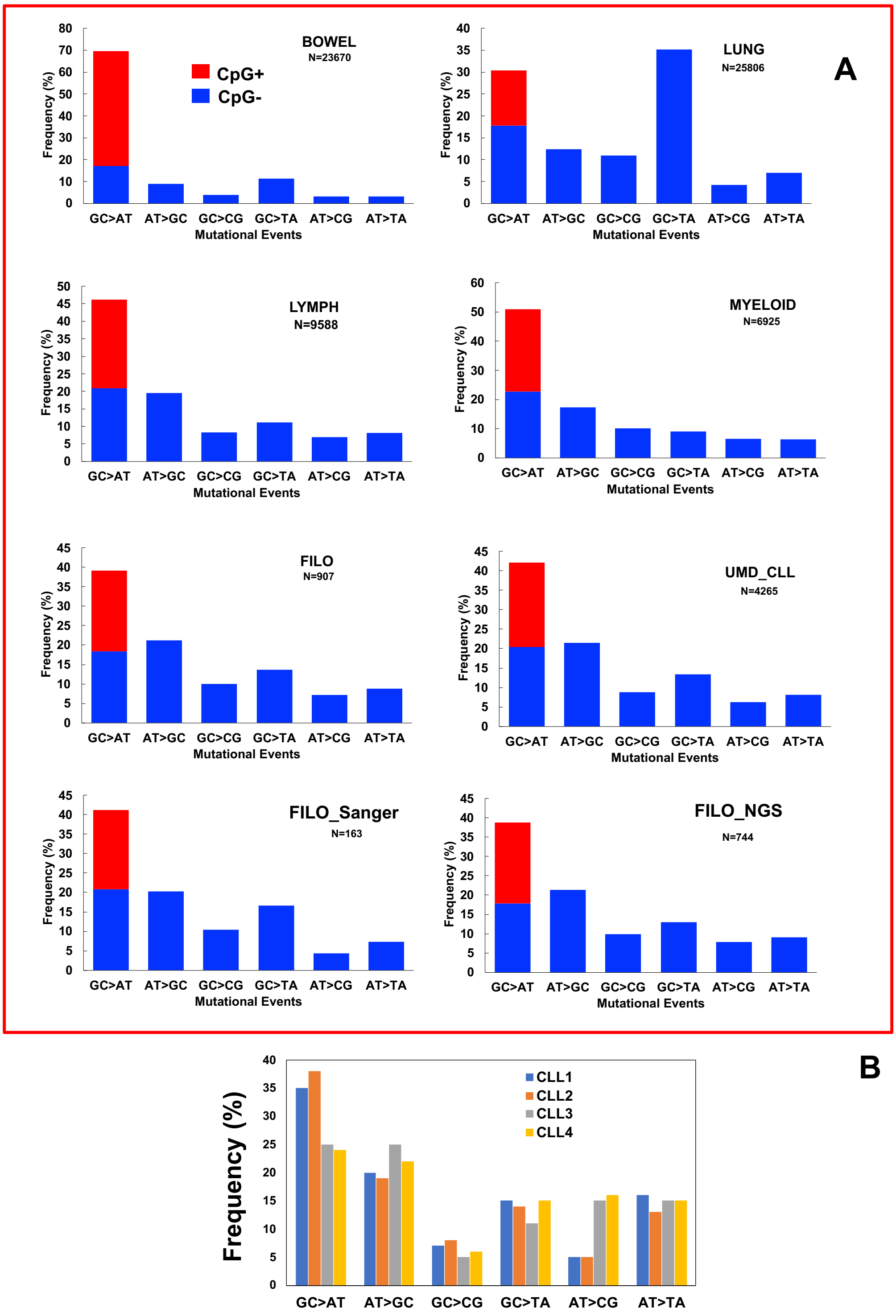

Supplementary Figure S2: High frequency of AT>GC transition in CLL.

A: Mutation spectra were obtained from the UMD_TP53 database for solid tumors (BOWEL or LUNG) or lymphoid and myeloid neoplasm (LYMPH or MYELOID). CpG+: mutations detected at CpG dinucleotides. CpG-: mutations detected outside CpG dinucleotides. Organ site definition for all tumors in UMD was performed using the OncoTree classification system [1]. CpG+:

B: Profile of somatic mutations in four CLL patients (CLL1 to 4) analyzed by whole genome sequencing [2].

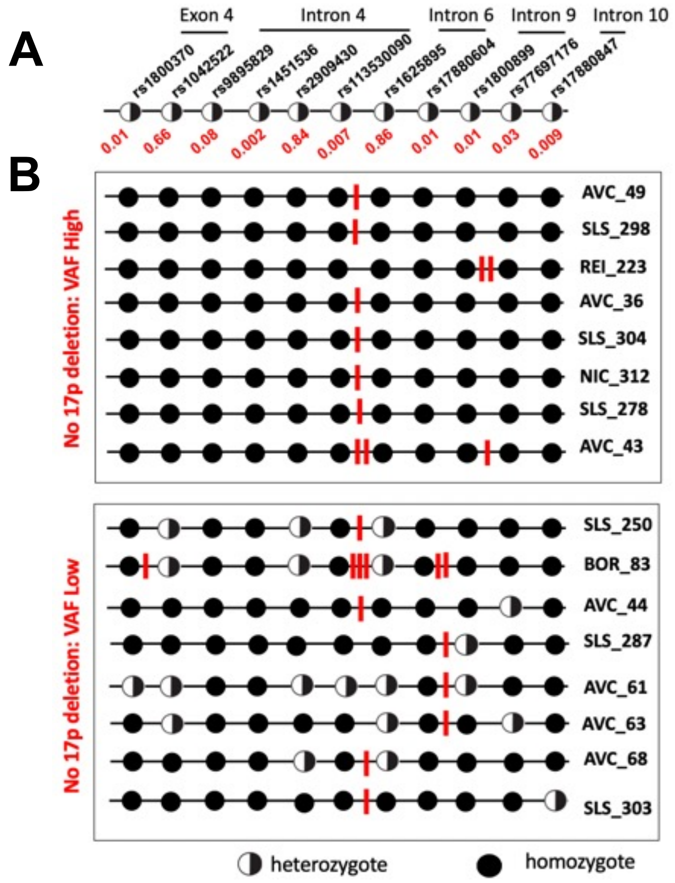

Supplementary Figure S3: Copy-neutral LOH (CN-LOH) is frequent in CLL and can be validated by SNP analysis.

A: Distribution of 11 *TP53* SNPs located in the region covered by NGS sequencing. For each SNP, the population frequency is shown in red. **B**: Haplotype of patients without 17p deletion with high (top) or low (bottom) VAF for *TP53* mutation. For each patient, the approximate position of the *TP53* variant is shown as a red bar.

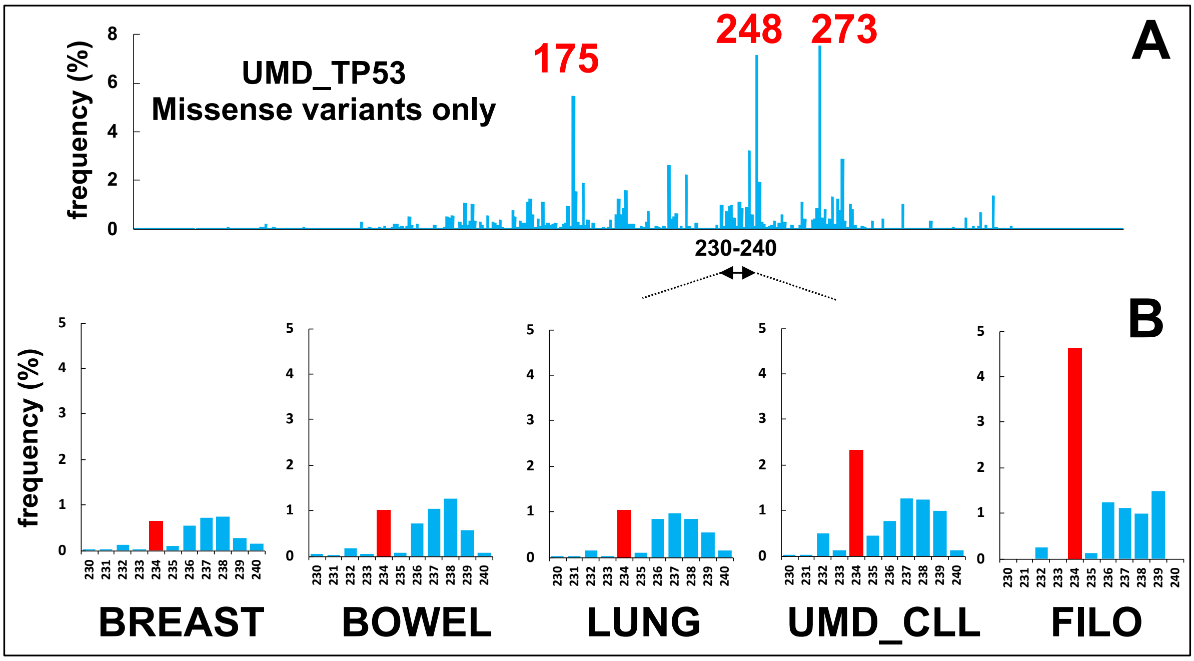

Supplementary Figure S4: Identification of *TP53* mutation hotspots in CLL in codon 234.

A: Distribution of mutations at each codon of the *TP53* protein. Only single nucleotide substitutions are analyzed. Classical *TP53* hotspot mutations are indicated in red with codons 175, 248 and 273 found in every type of cancer. **B**: Close up from figure A for codons 230 to 240 for different types of cancer. Data were updated from the work of Lazarian *et al*. with new patients from the FILO cohort [3].

A

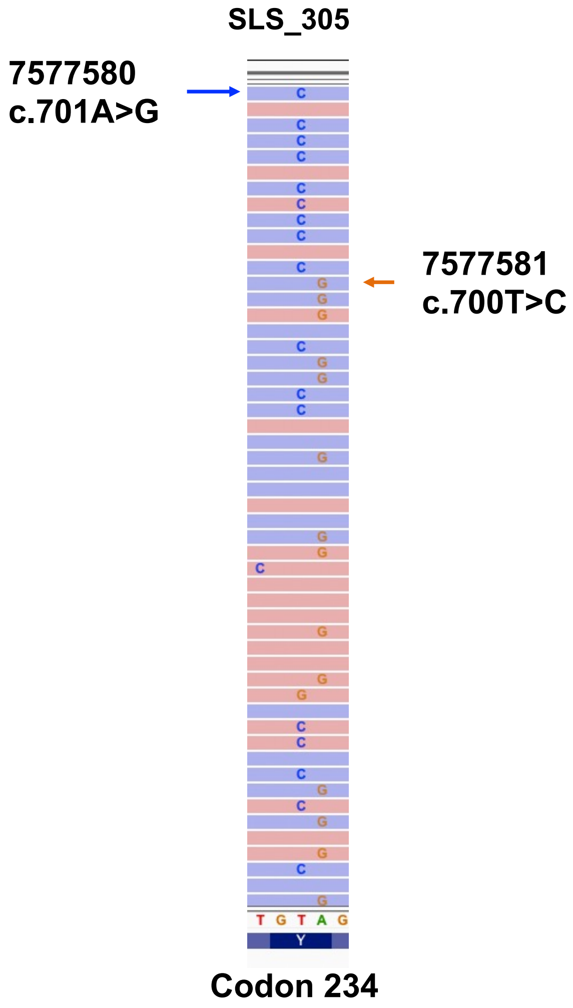

B

LRG_321t1	TP53alpha	Disease_name	Sample_ID	Complexity	Authors	Year	Journal	Treatment
c.701A>G	p.Tyr234Cys	CLL	SLS_305	MM	Present work	NR	NR	Multiple lines of treatment including CLB
c.700T>C	p.Tyr234His	CLL	SLS_305	MM	Present work	NR	NR	Multiple lines of treatment including CLB
c.701A>G	p.Tyr234Cys	CLL	nAVC_129	MM	Present work	NR	NR	Multiple lines of treatment including CLB
c.700T>C	p.Tyr234His	CLL	nAVC_129	MM	Present work	NR	NR	Multiple lines of treatment including CLB
c.701A>G	p.Tyr234Cys	CLL	CLL-NHLBI-0011	MM	Landau DA, et al.	2017	Nat Commun	Treated; no specific information
c.701A>C	p.Tyr234Ser	CLL	CLL-NHLBI-0011	MM	Landau DA, et al.	2018	Nat Commun	Treated; no specific information
c.701A>G	p.Tyr234Cys	CLL	8	MM	Malcikova J, S et al.	2015	Leukemia	Multiple lines of treatment including CLB
c.701A>C	p.Tyr234Ser	CLL	8	MM	Malcikova J, S et al.	2015	Leukemia	Multiple lines of treatment including CLB
c.701A>G	p.Tyr234Cys	CLL	485	MM	Malcikova J, S et al.	2015	Leukemia	Multiple lines of treatment including CLB
c.701A>C	p.Tyr234Ser	CLL	485	MM	Malcikova J, S et al.	2015	Leukemia	Multiple lines of treatment including CLB
c.700T>C	p.Tyr234His	CLL	485	MM	Malcikova J, S et al.	2015	Leukemia	Multiple lines of treatment including CLB
c.701A>G	p.Tyr234Cys	AML	AML_547	DMU	Metzeler KH, et al.	2016	Blood	Multiple lines of treatment: no alkylating agents
c.700T>C	p.Tyr234His	AML	AML_547	DMU	Metzeler KH, et al.	2016	Blood	Multiple lines of treatment: no alkylating agents

Supplementary Figure S5: Multiple *TP53* variants in codon 234 in a same patient are associated with CLB exposure.

A: IGV plot demonstrates the phasing of patient SLS_205 with multiple *TP53* variants in codon 234. Individual DNA sequencing reads harboring mutations within the same exon and no more than 50 nucleotides apart were analyzed. **B**: Details for the six patients harboring multiple mutations in codon 234 included in the entire UMD_TP53 database.

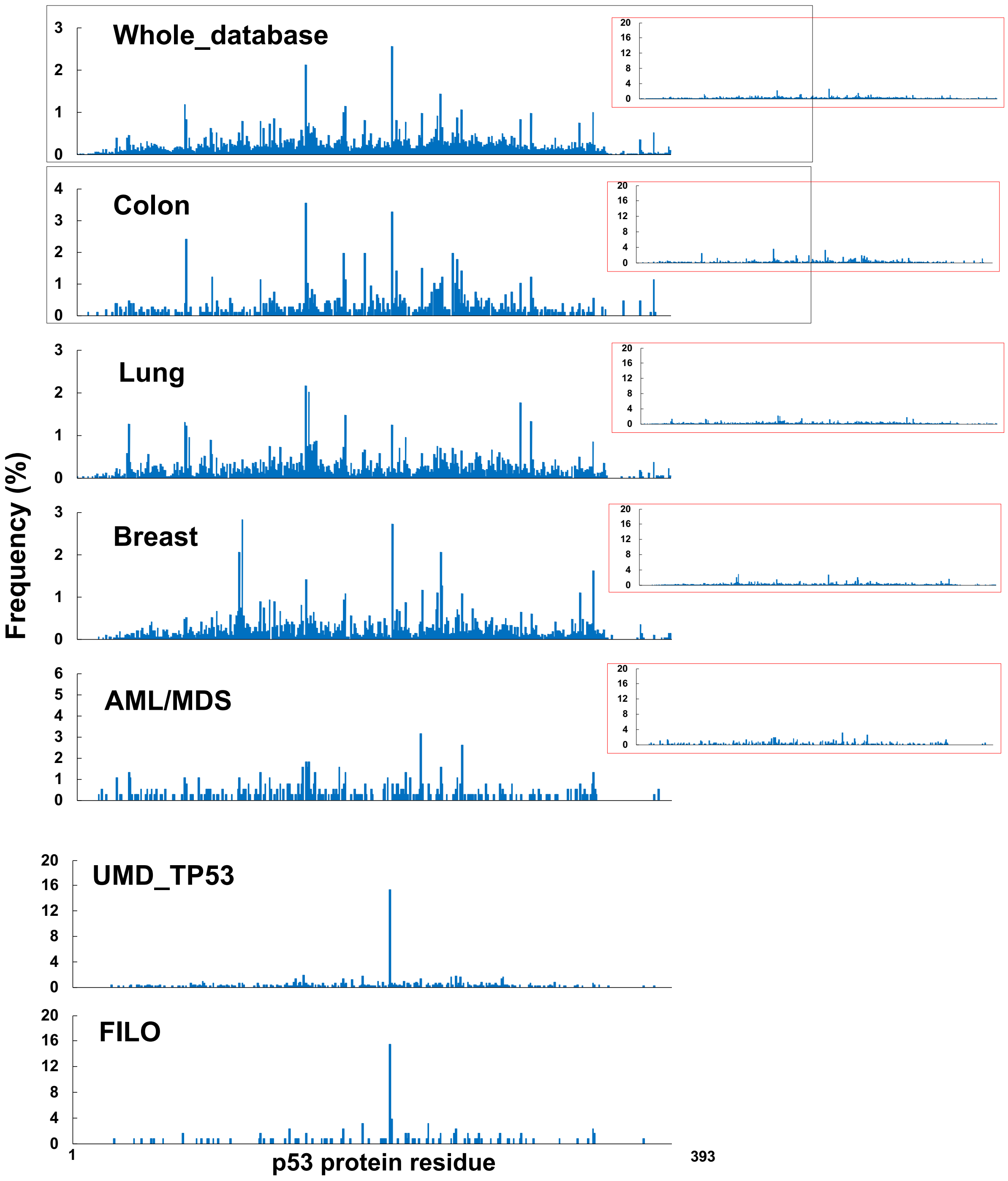

Supplementary Figure S6: Variant NM_000546_c.626_627del (NP_000537_p.Arg209LysfsTer6) is a hotspot mutation in CLL.

Distribution of frameshift mutations at each codon of the *TP53* protein in various types of cancer. Only insertions and deletions are analyzed. Scales for the distributions are not standardized. The insets for non-chronic lymphocytic leukemia have been standardized to chronic lymphocytic leukemia data to emphasize the high frequency of variant NM_000546_c.626_627del. AML/MDS: acute myeloid leukemia and myelodysplastic syndrome. Lung: NSCLC and SCLC.

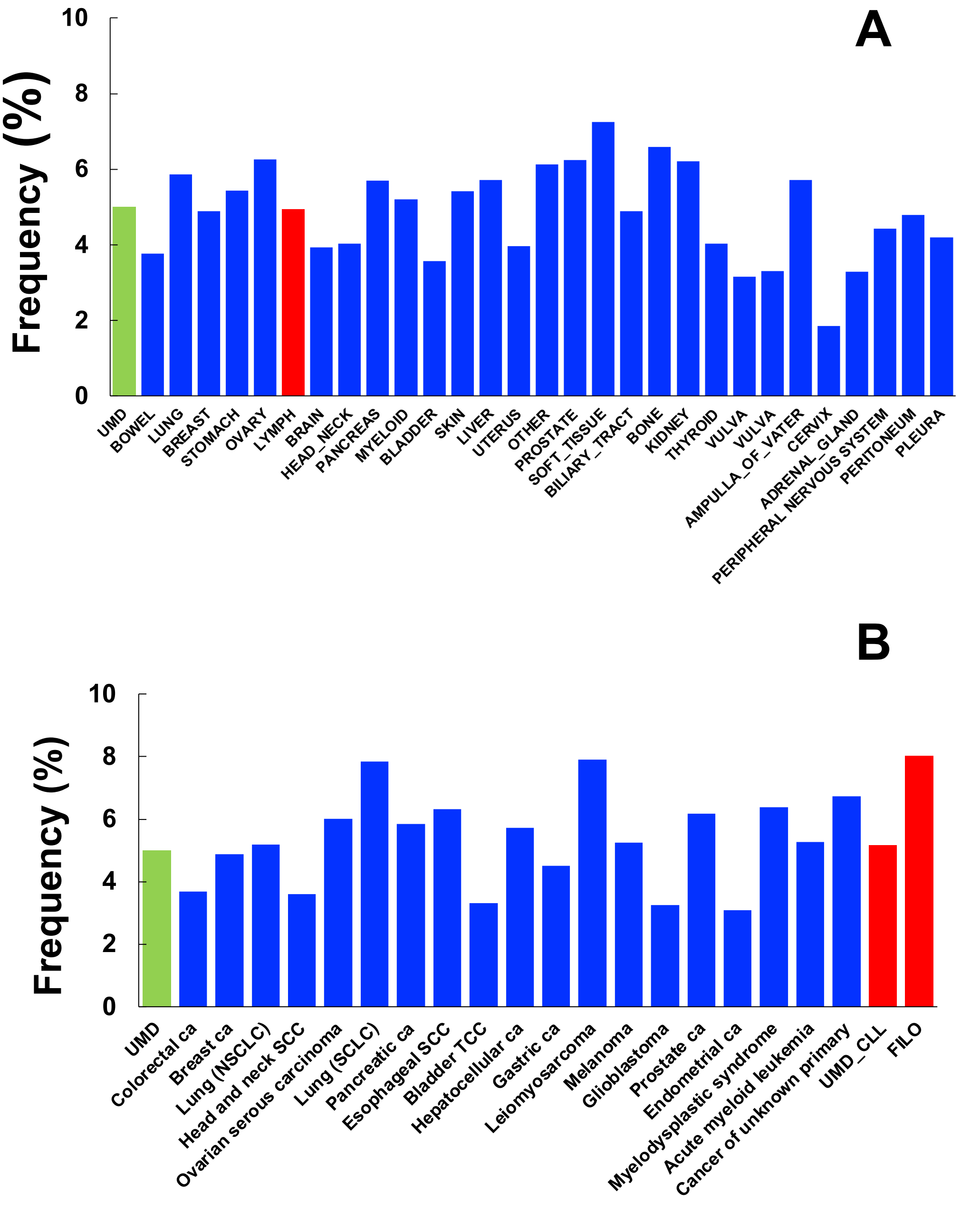

Supplementary Figure S7: Frequency of splice mutations in the *TP53* gene among the various types of cancer.

A: Cancer types were classified according to organ sites as defined in OncoTree. LYMPH: class including all CLL patients (5795) as well as other lymphoid cases (5392). **B**: Cancer types were classified according to tumor types.

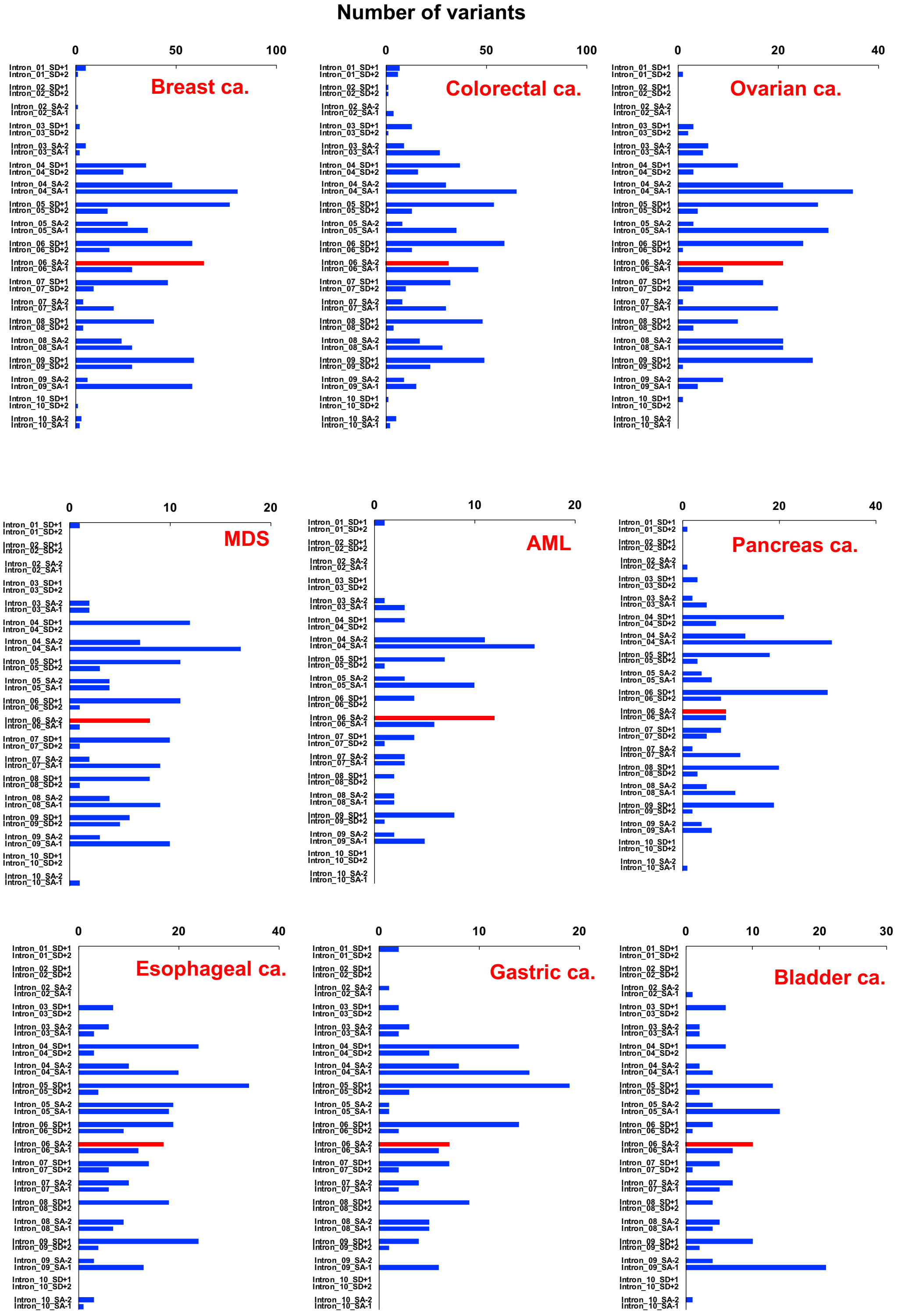

Supplementary Figure S8: CLL hotspot mutation in the splice acceptor signal of intron 6.

Distribution of mutations in the 20 splice signals of the 10 introns of the *TP53* gene for various types of cancer. Data are taken from the most recent release of UMD_TP53.

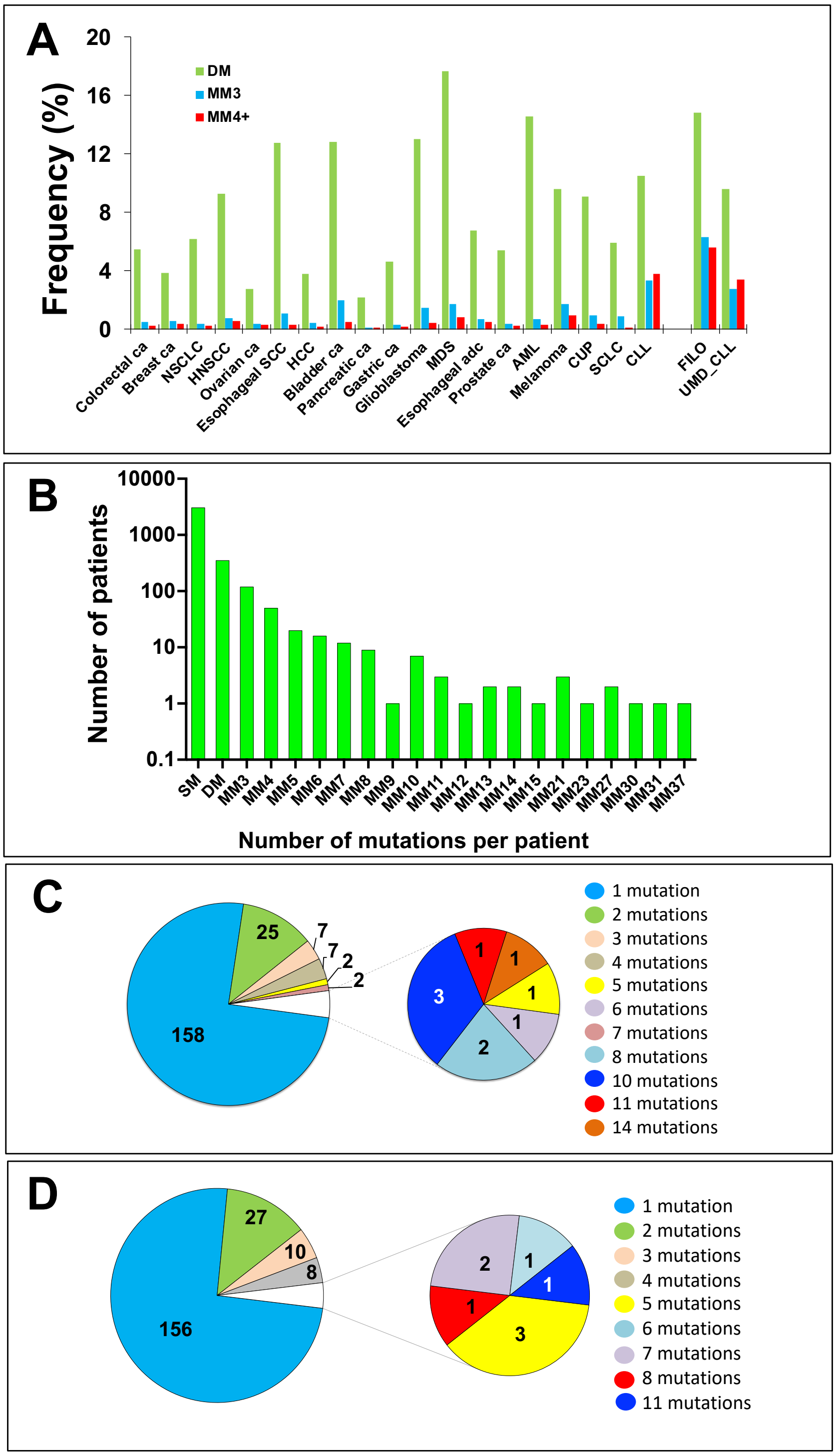

Supplementary Figure S9: Wide variant distribution in the number of *TP53* variants in CLL patients.

A: *TP53* cancer types were analyzed for tumors carrying two (DM), three (MM3) or more than three (MM4+) *TP53* variants. CLL tumors were split into two subgroups including either FILO or UMD_CLL patients. **B**: Number of mutations per patient. SM: patients with a single mutation; DM: patients with two *TP53* variants: MMn: multi-mutated patients with n mutations. **C and D**: Distribution of the number of mutations in tumors from the studies of Catherwood *et al*. and Bomben *et al*. [4, 5].

AVC_40

7578176
c.672+1G>T

7578223
c.626_627del

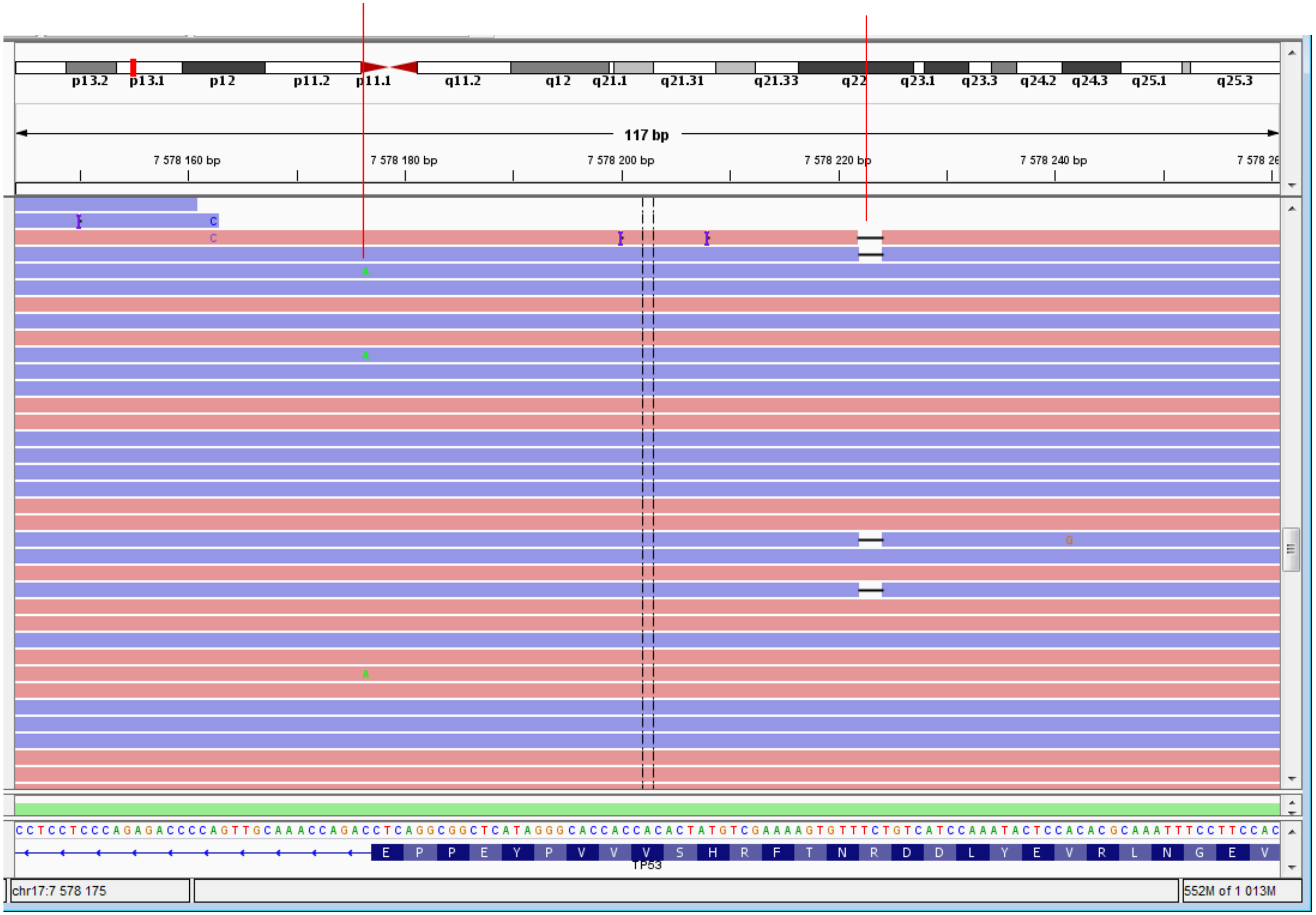

Supplementary Figure S10a

AVC_62

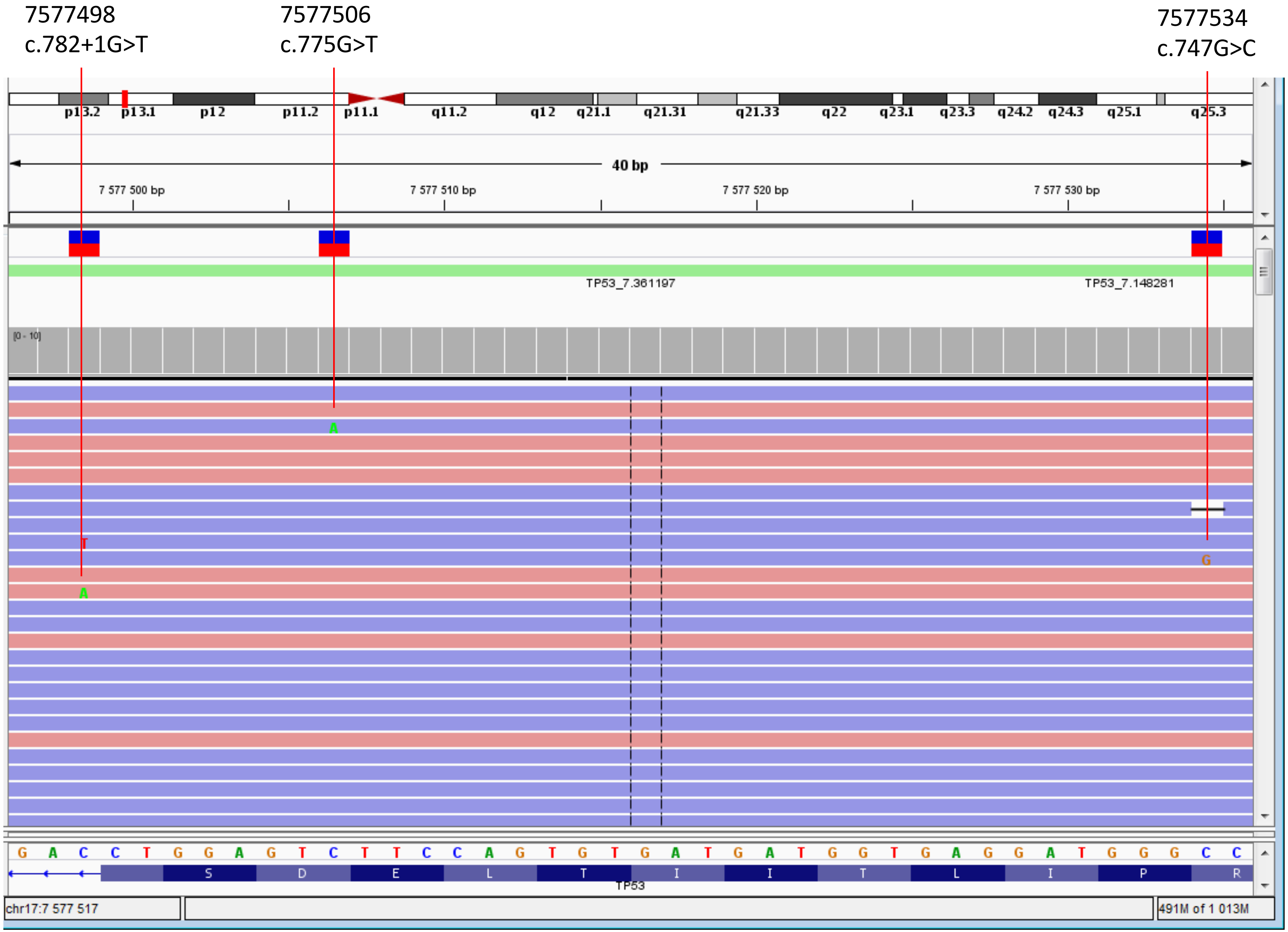

Supplementary Figure S10b

AVC_62

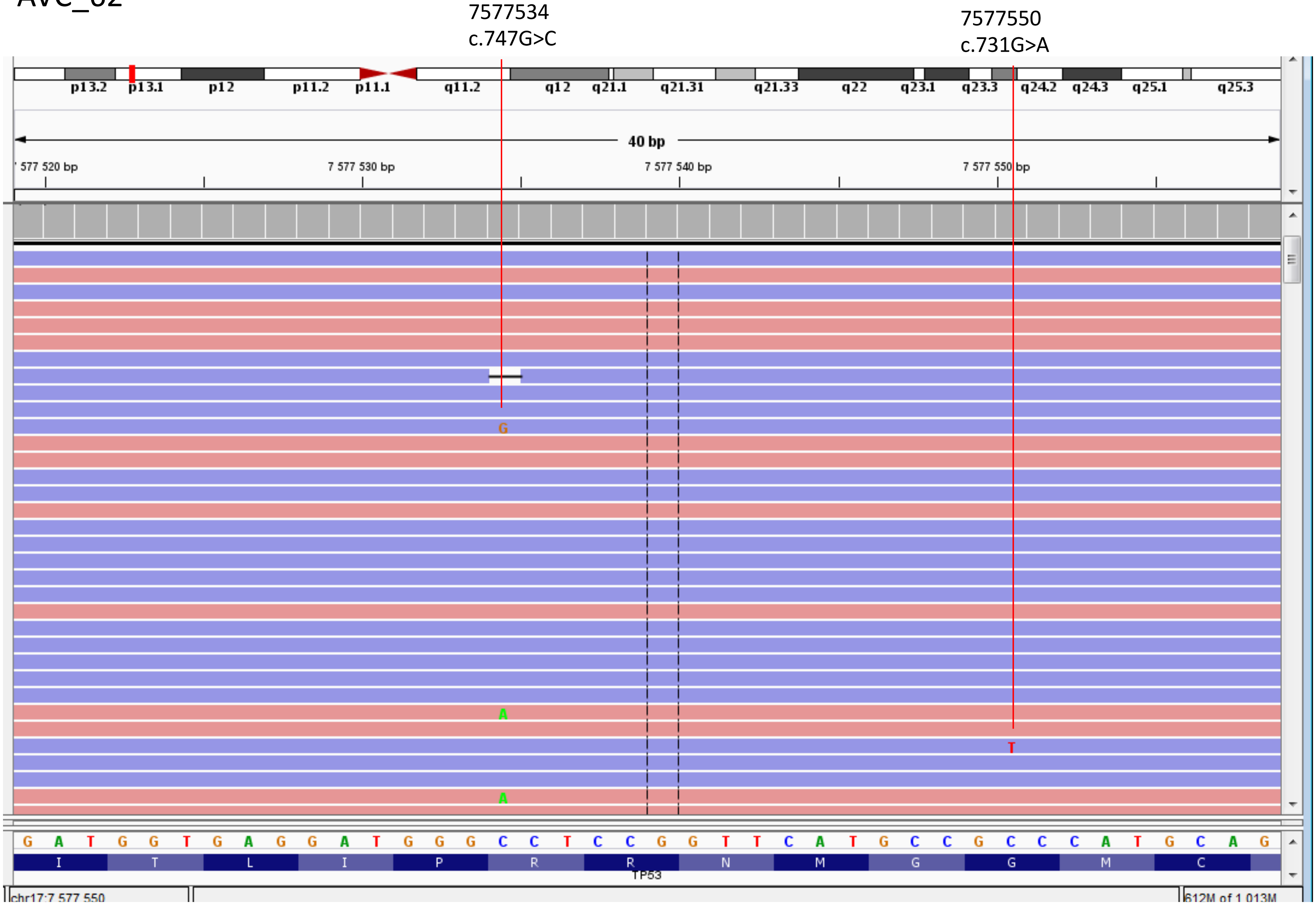

Supplementary Figure S10c

AVC_62

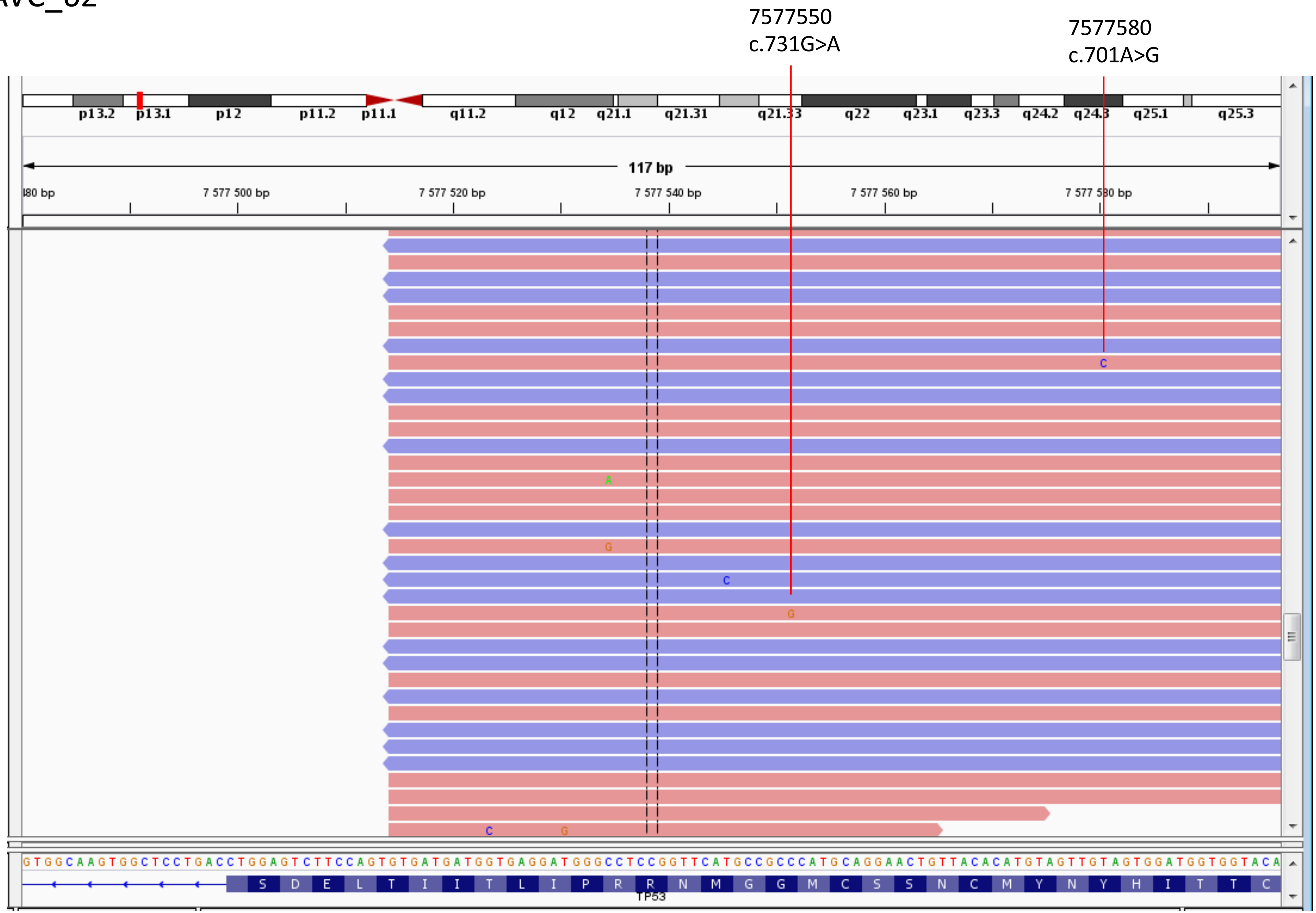

Supplementary Figure S10d

AVC_62

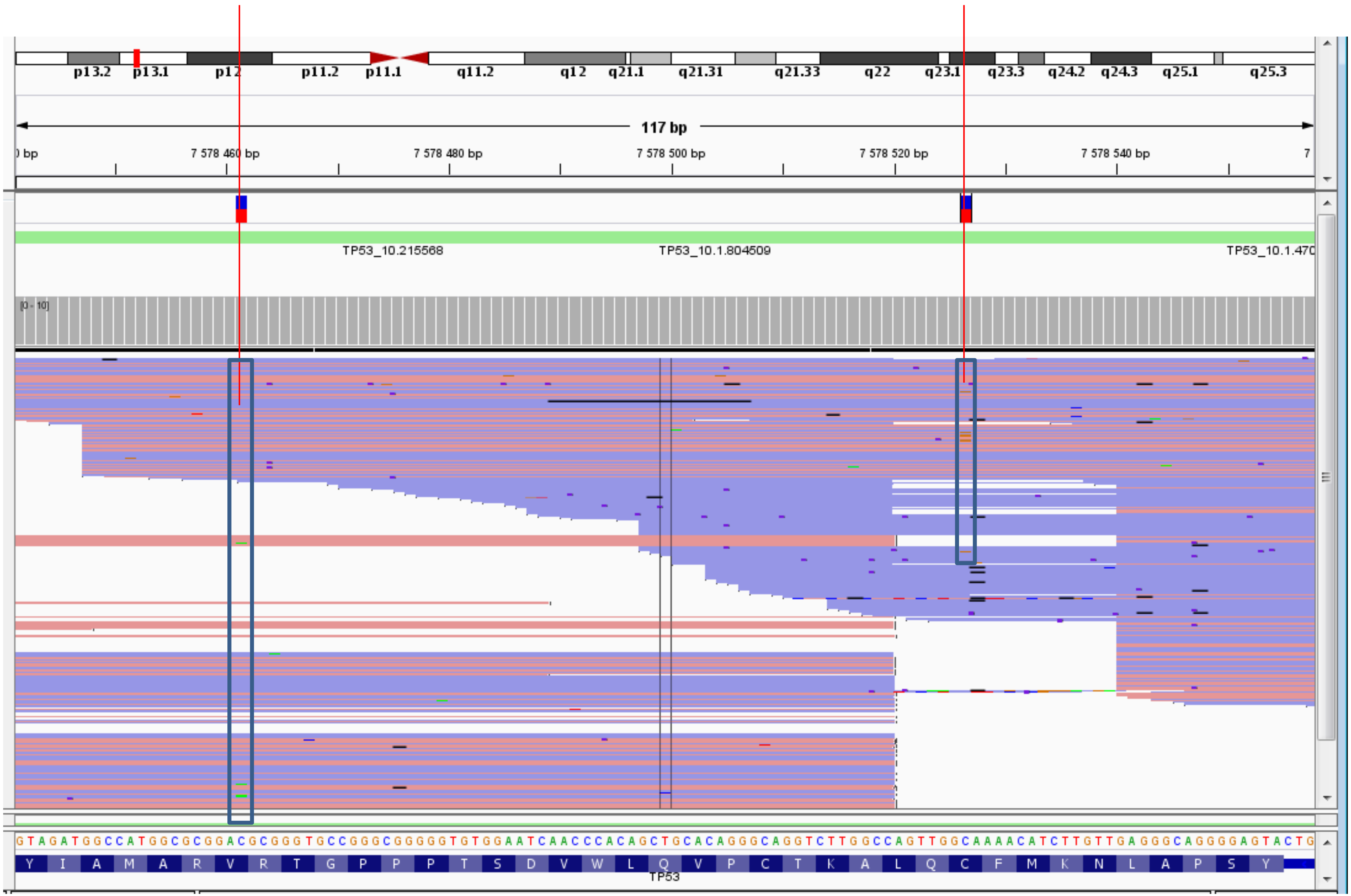

Supplementary Figure S10e

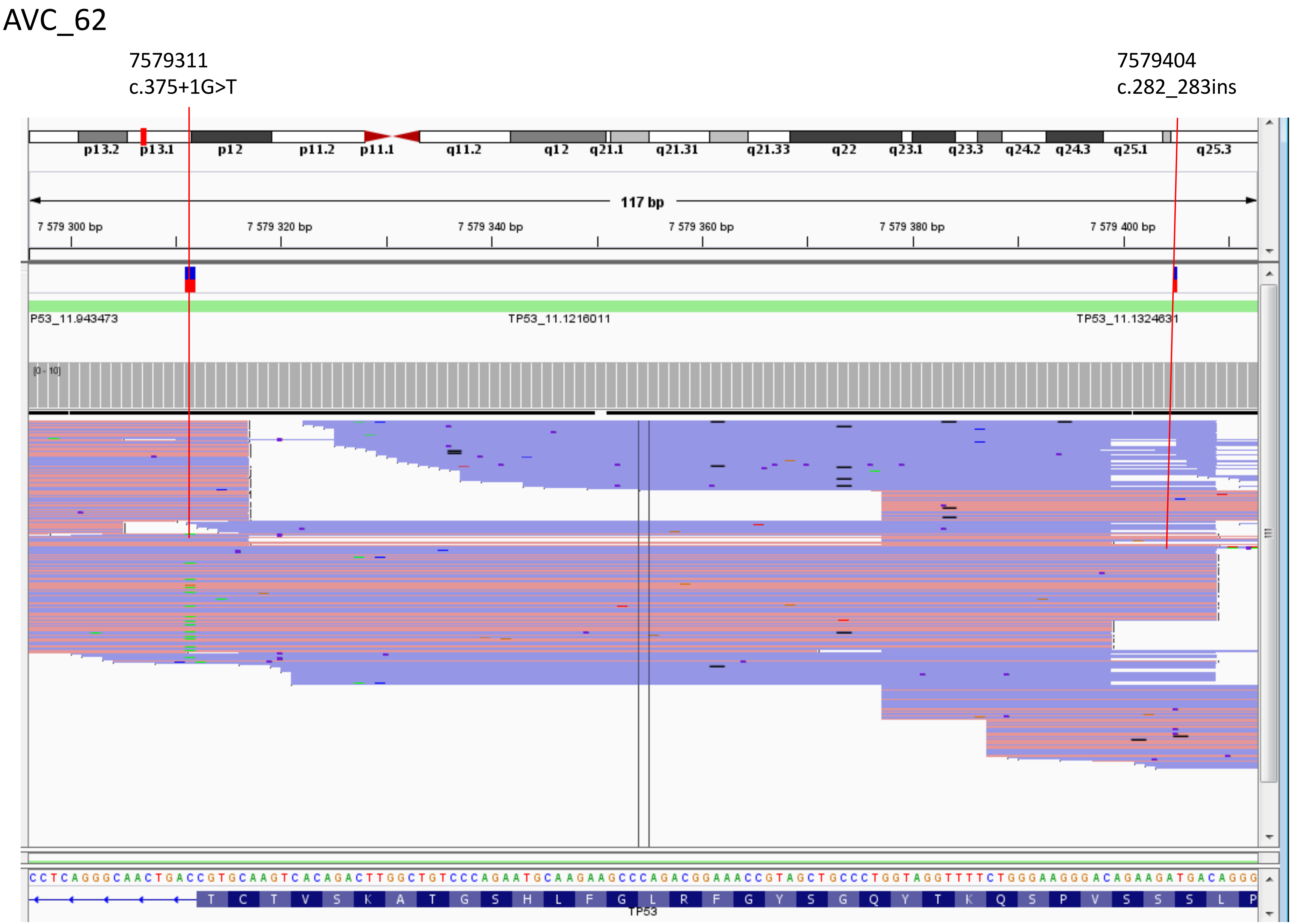

Supplementary Figure S10f

AVC_20

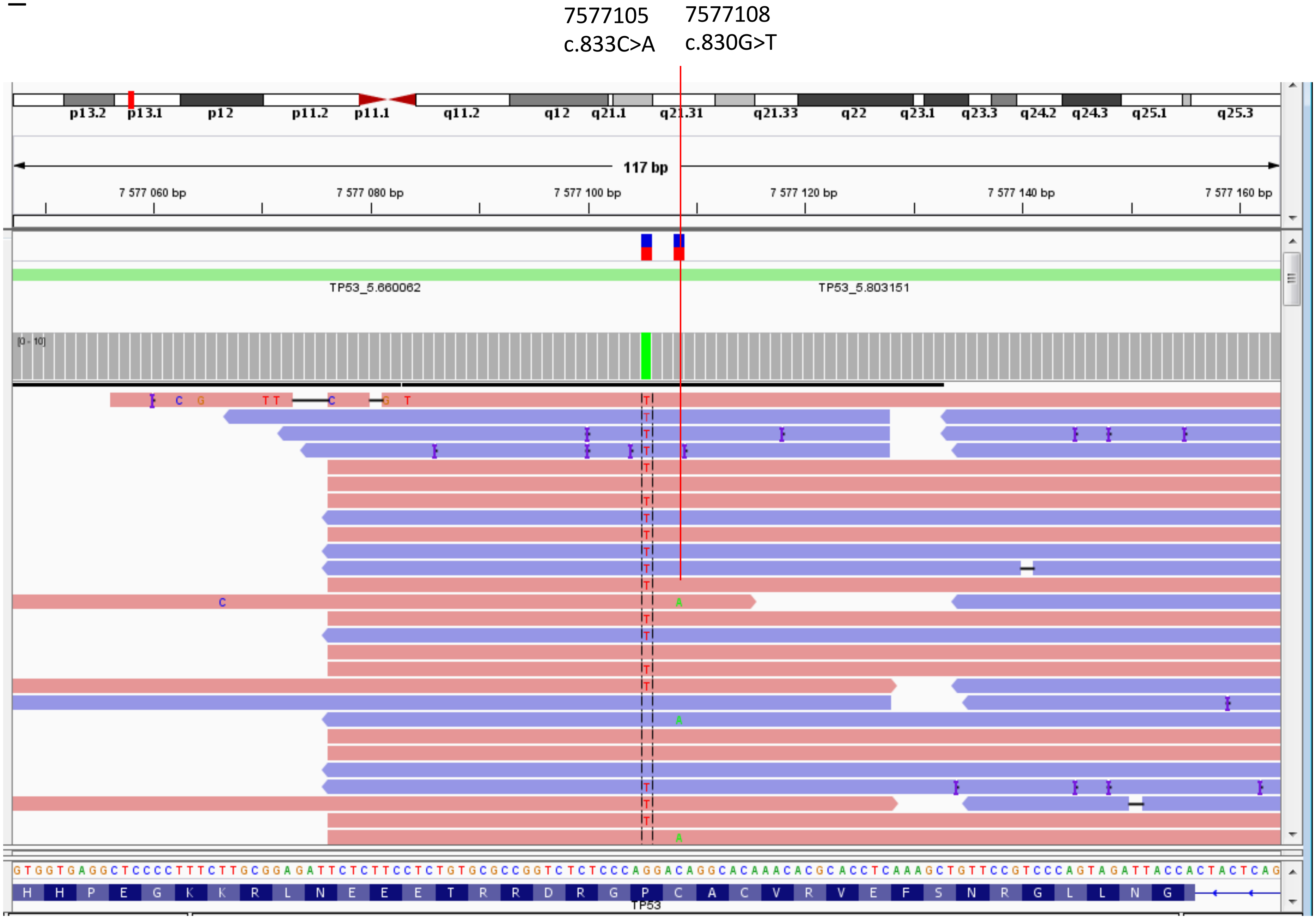

Supplementary Figure S10g

AVC_69

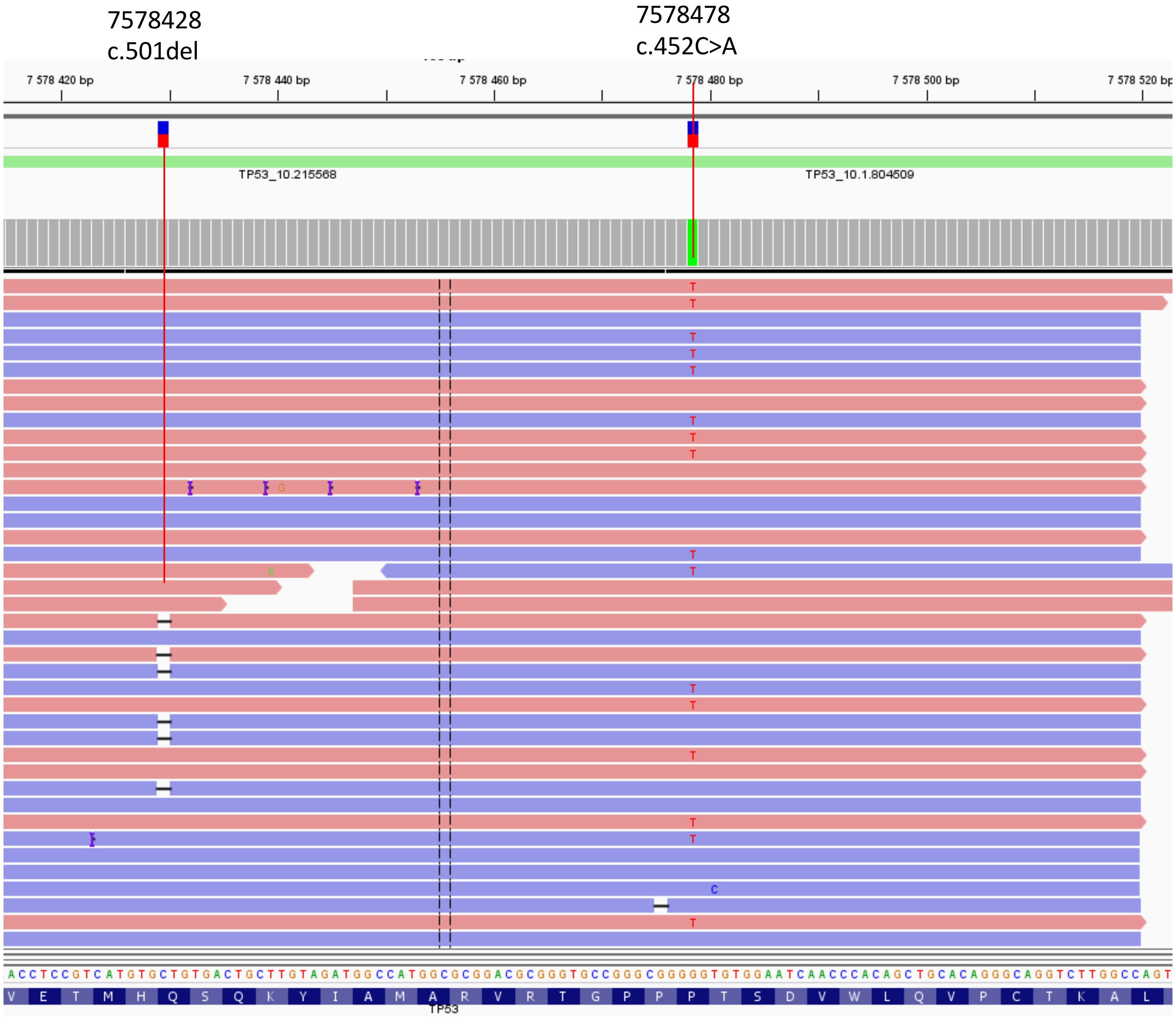

Supplementary Figure S10h

AVC_59

7577539
c742C>T

7577551
c.730G>T

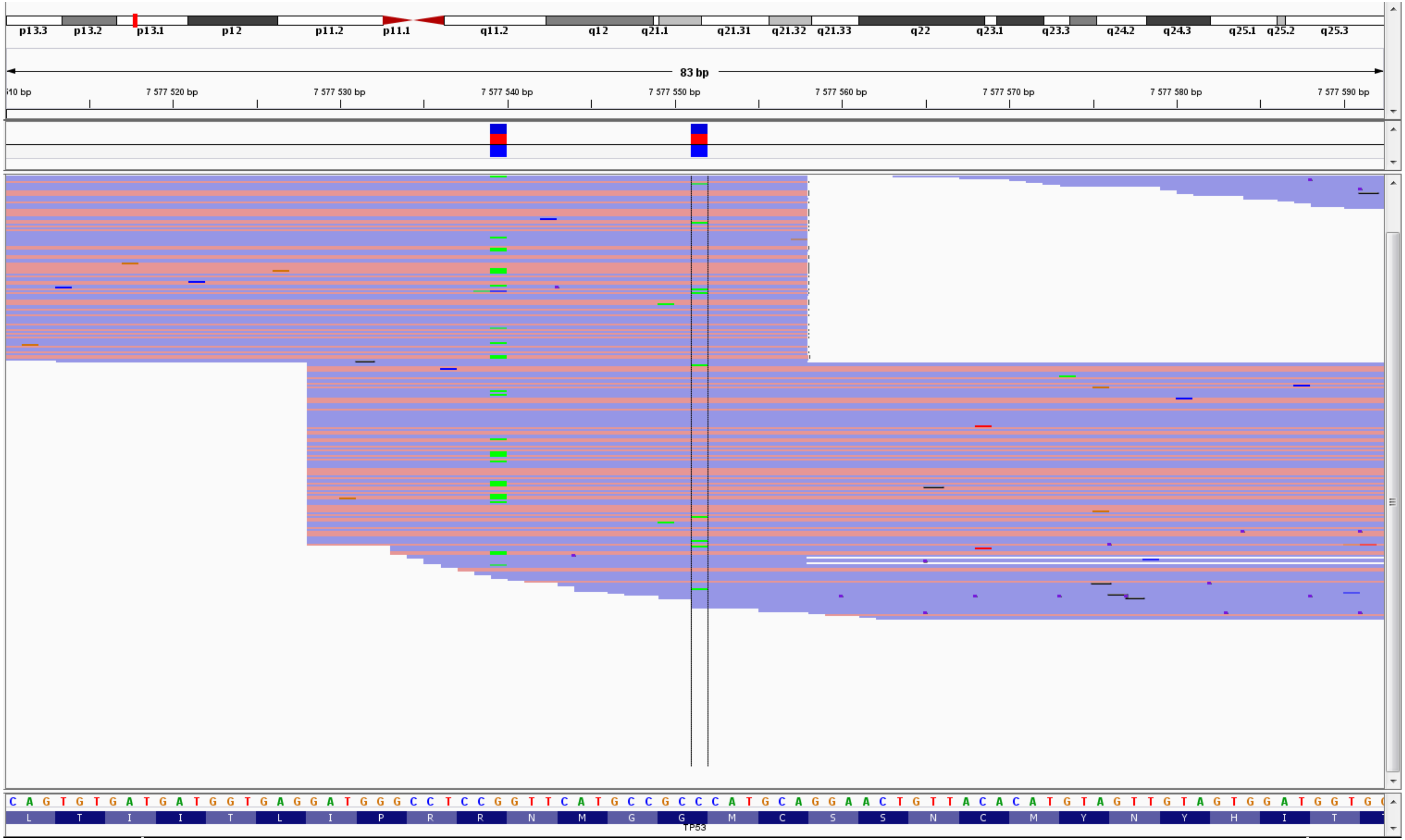

Supplementary Figure S10i

AVC_47

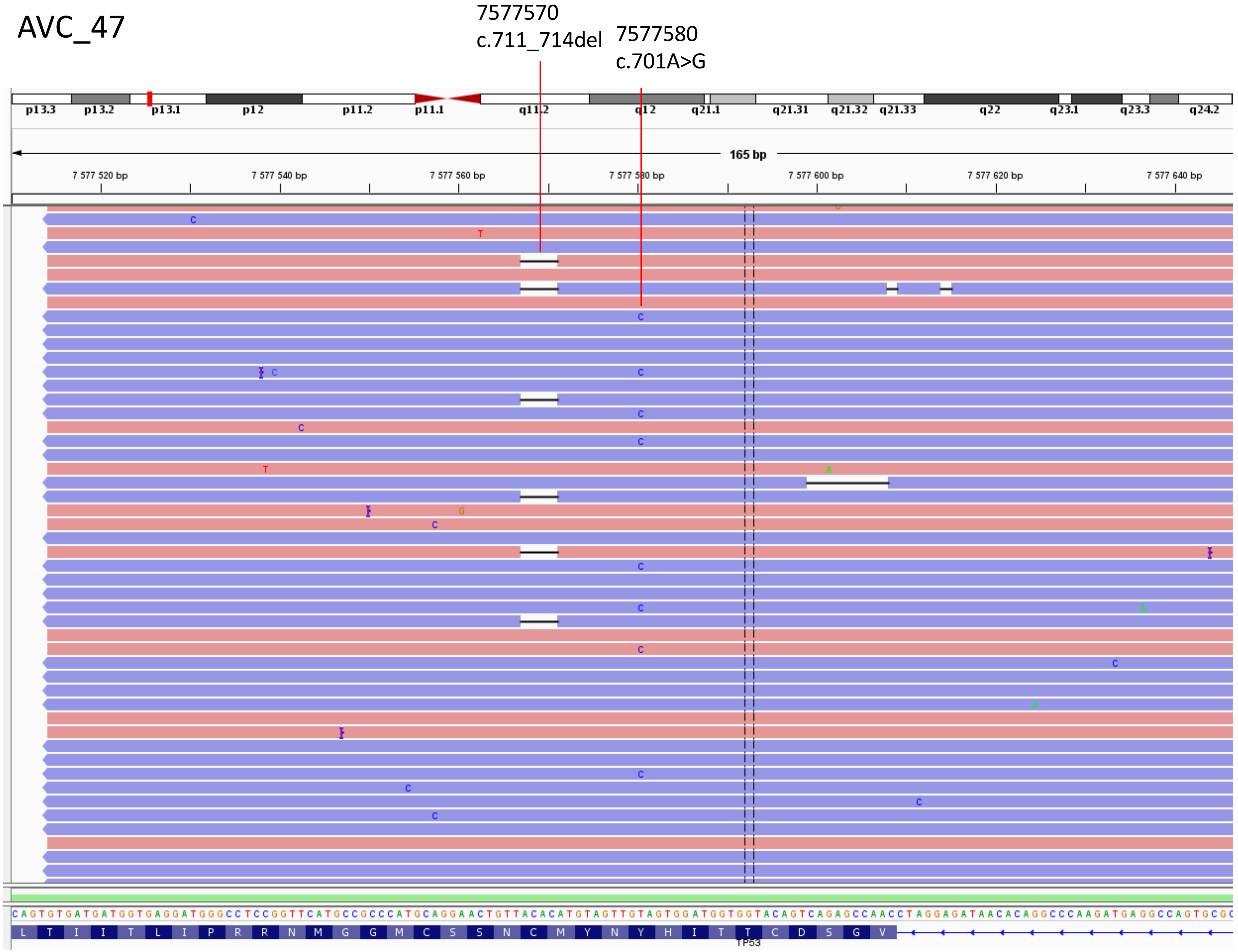

Supplementary Figure S10j

ANG_9

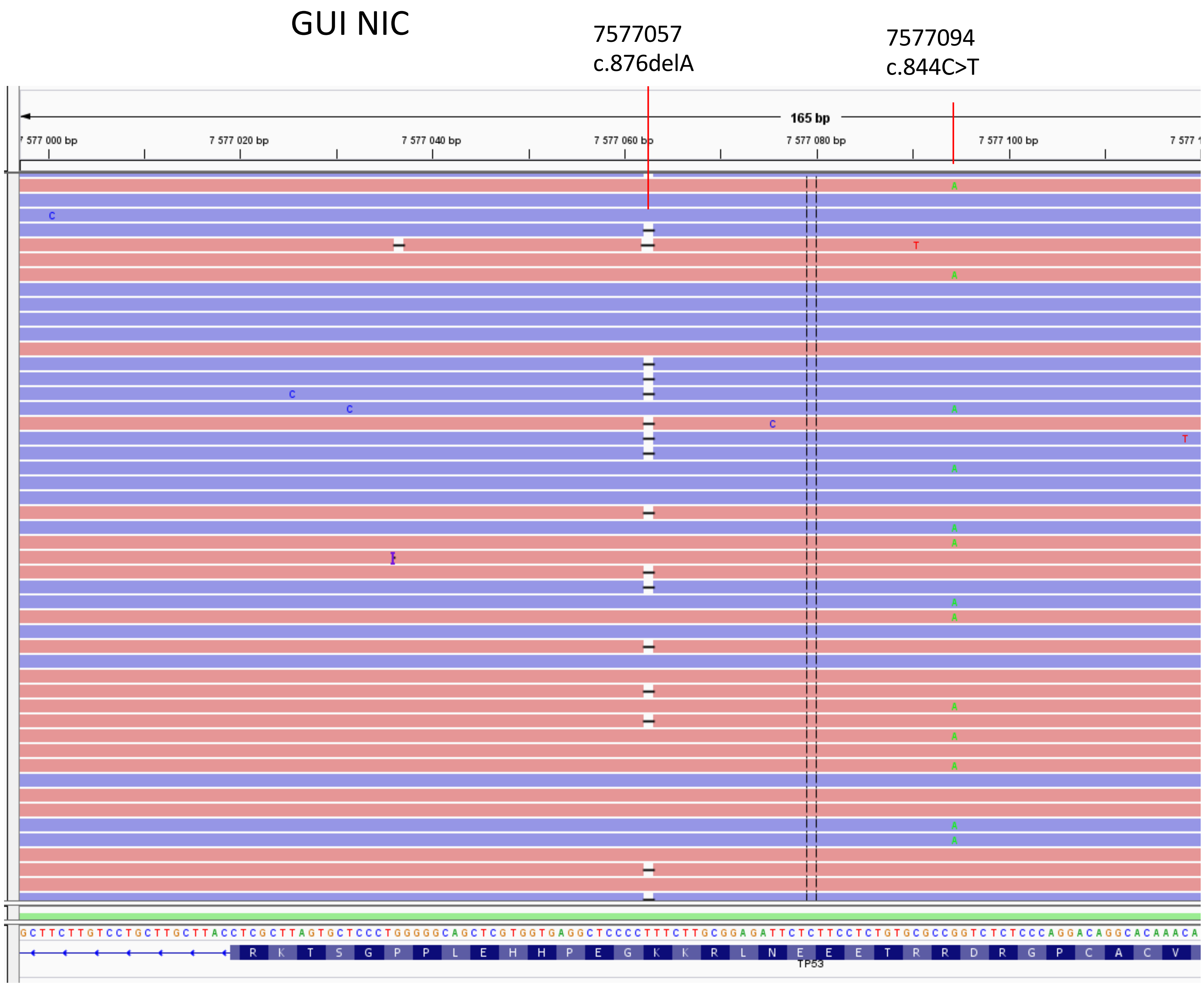

Supplementary Figure S10k

ANG_16

7578190
c.659A>G

7578265
c.584T>C

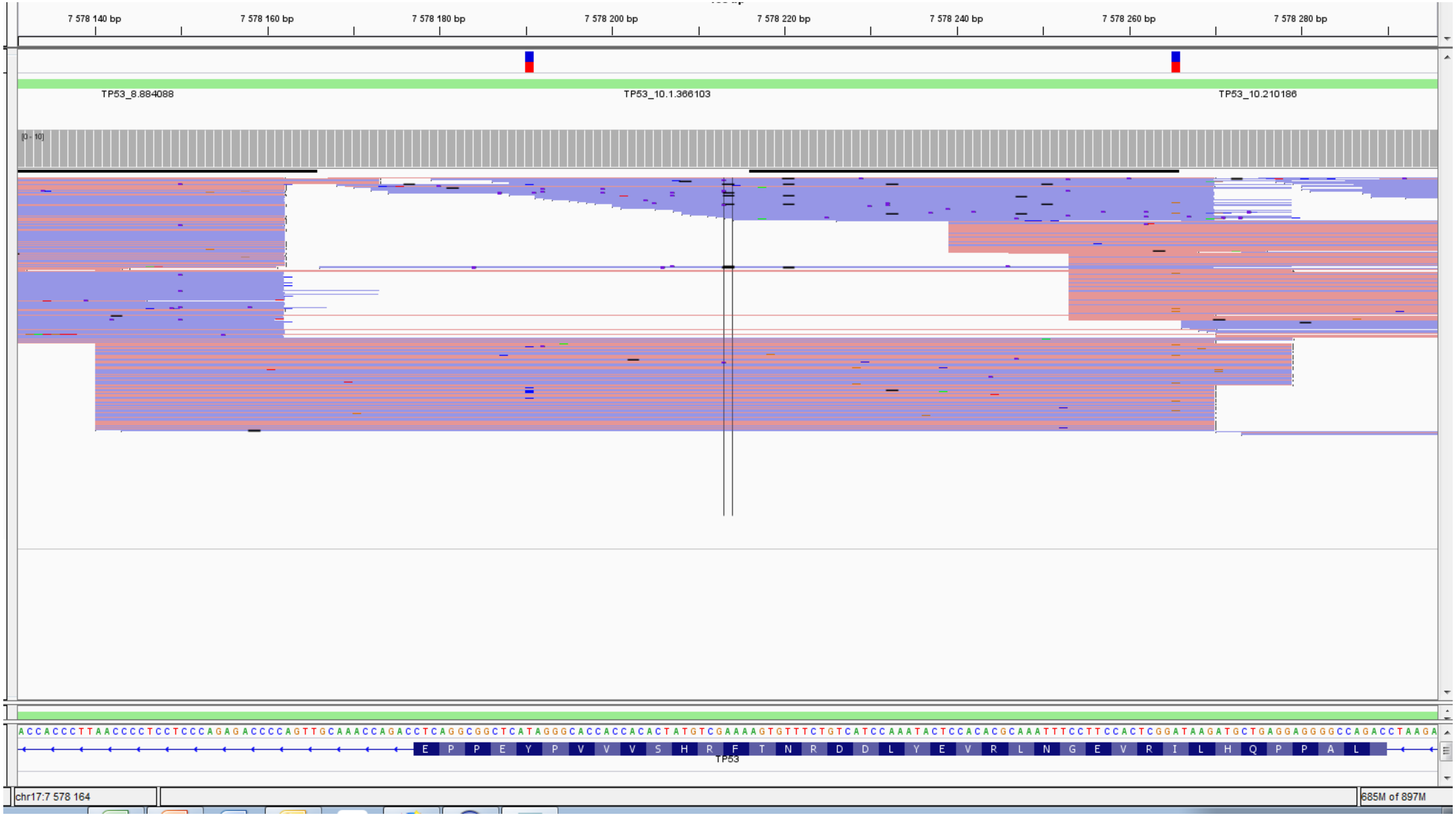

Supplementary Figure S10I

SLS_305

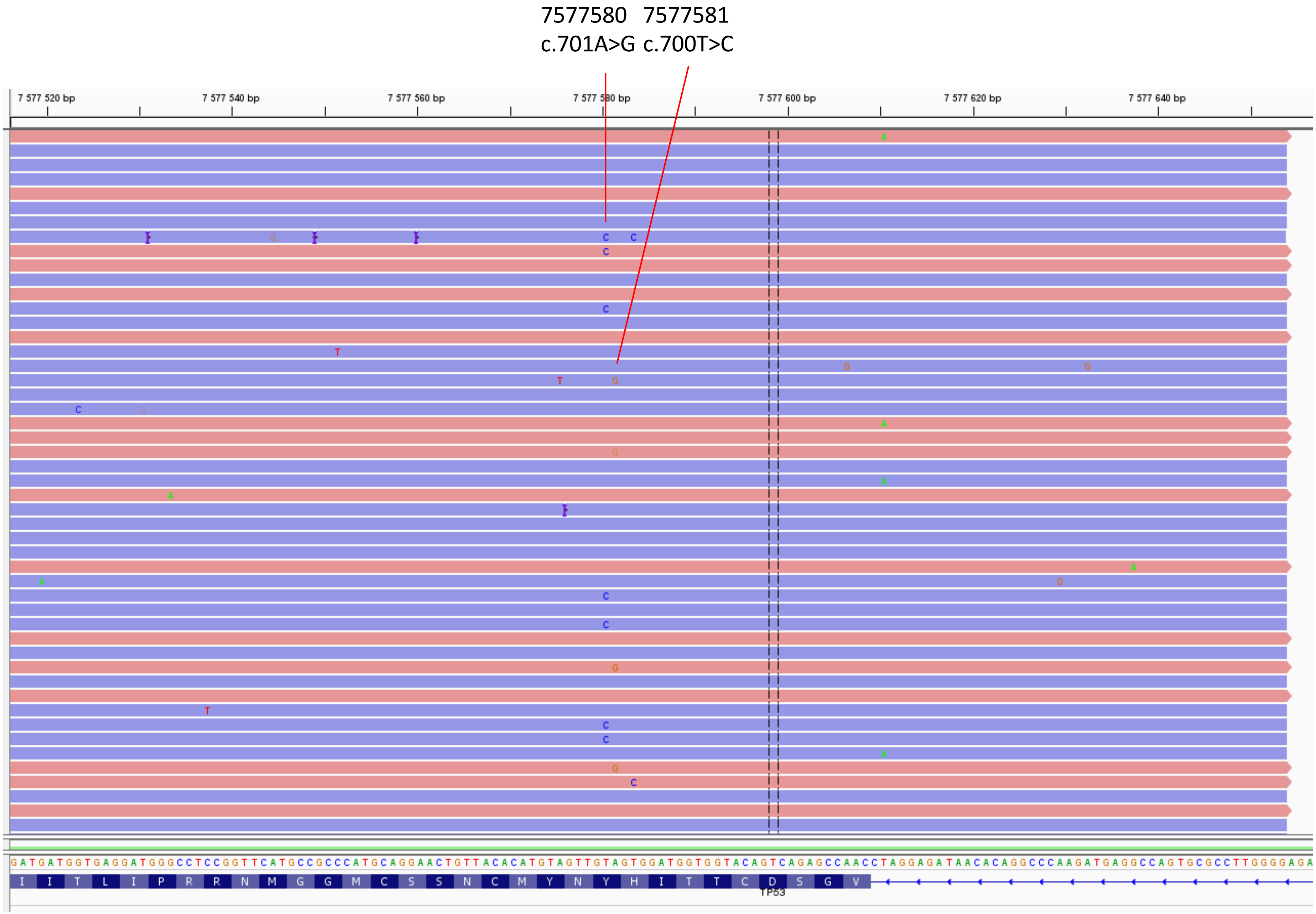

Supplementary Figure S10m

ANG_17

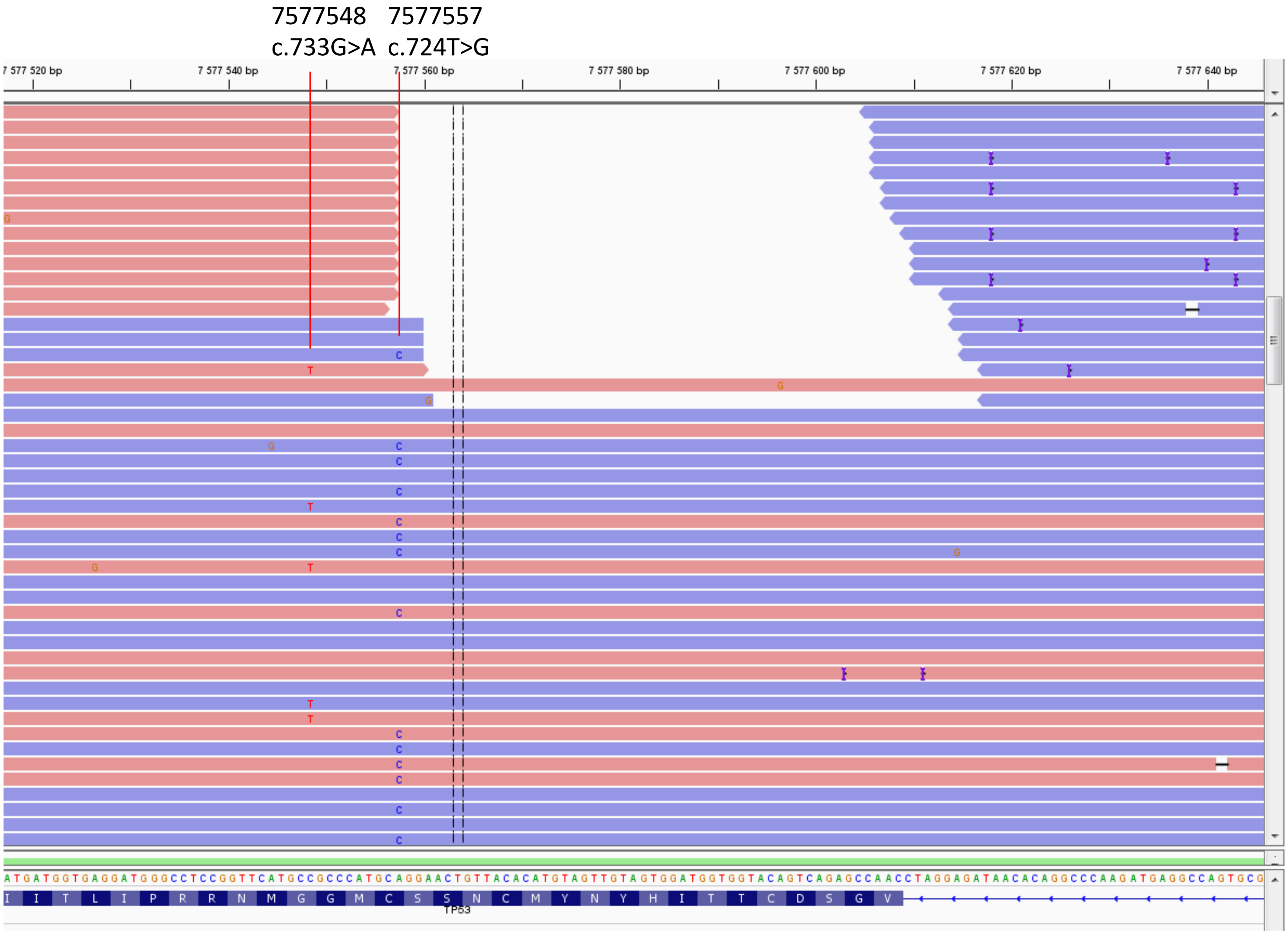

Supplementary Figure S10n

ANG_12

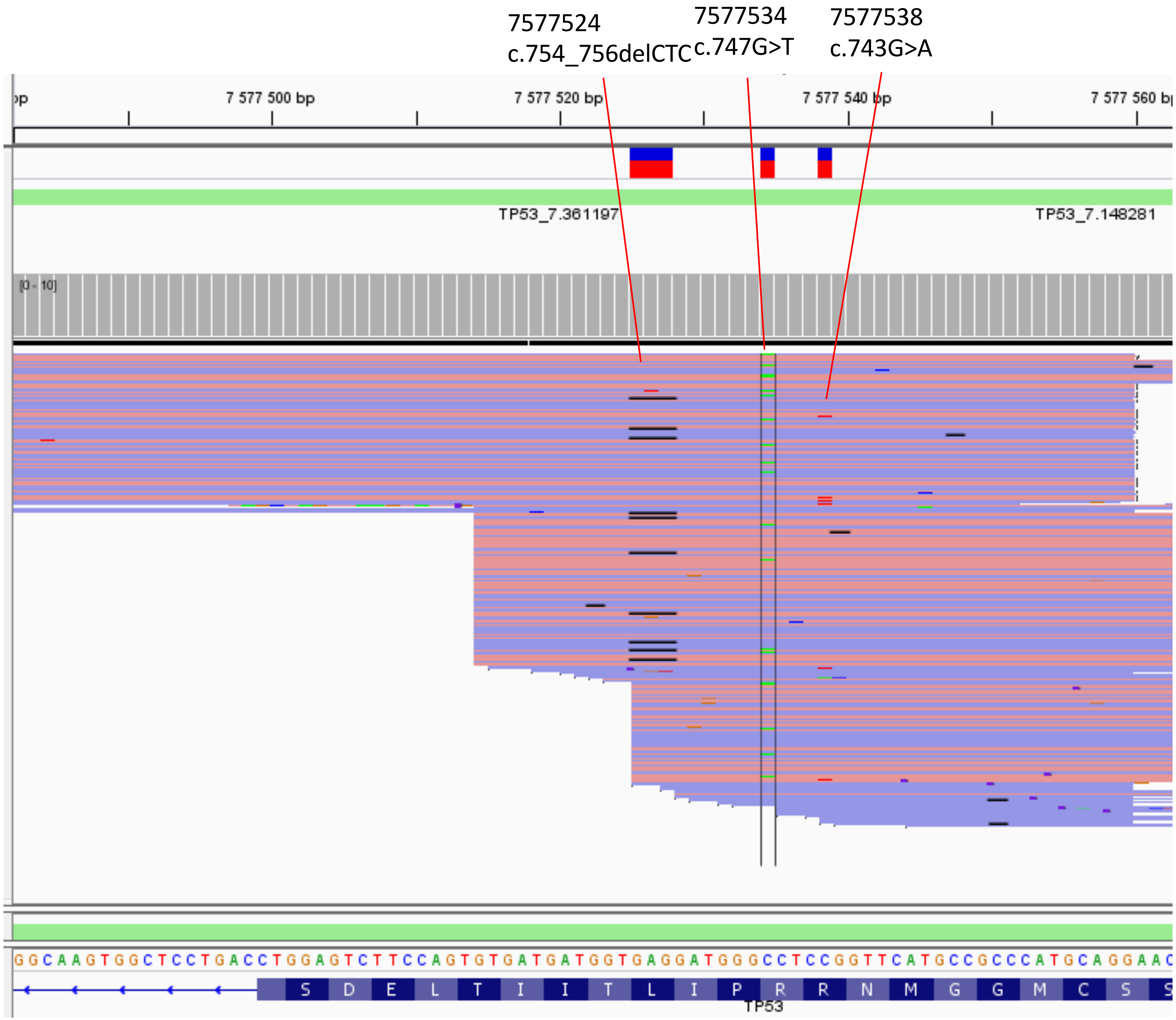

Supplementary Figure S10o

ANG_13

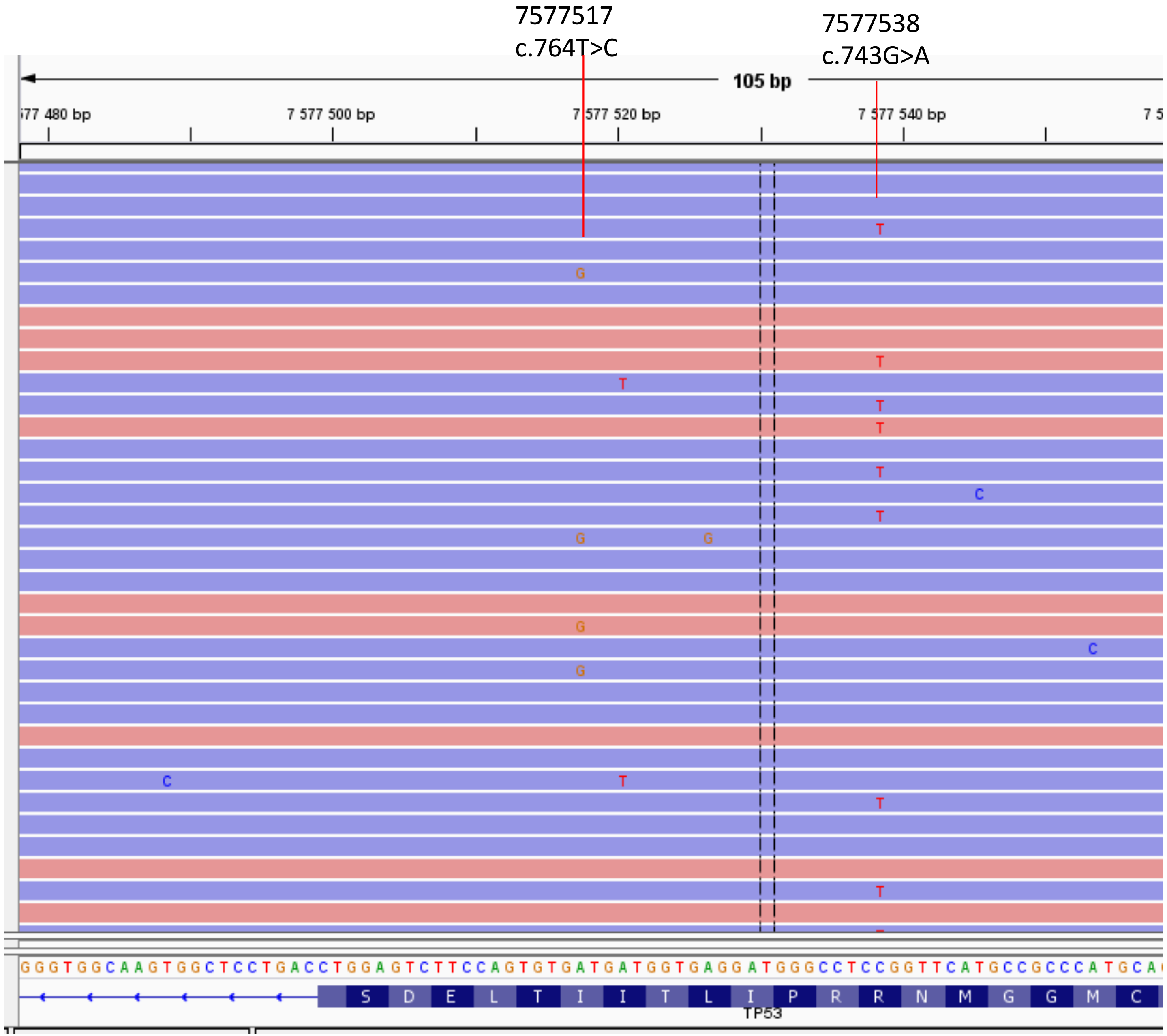

Supplementary Figure S10p

Supplementary Figure S10 to 10p: *TP53* mutations in FILO-cohort polymutated patients are in different alleles.

Each IGV plot depicts the phasing of patients with multiple *TP53* variants. Individual DNA sequencing reads harboring mutations within the same exon and no more than 50 nucleotides apart were analyzed.

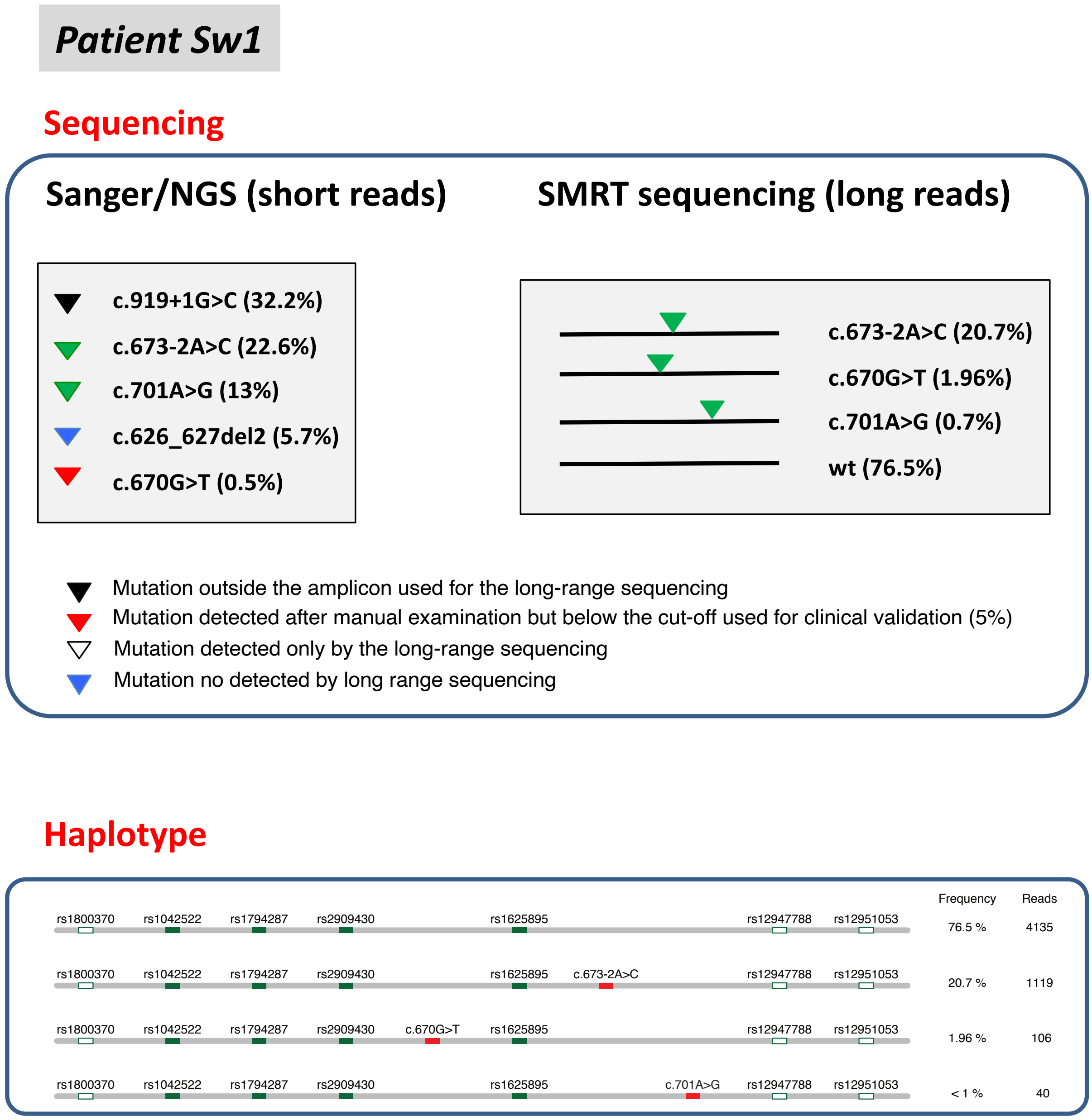

Supplementary Figure S11A

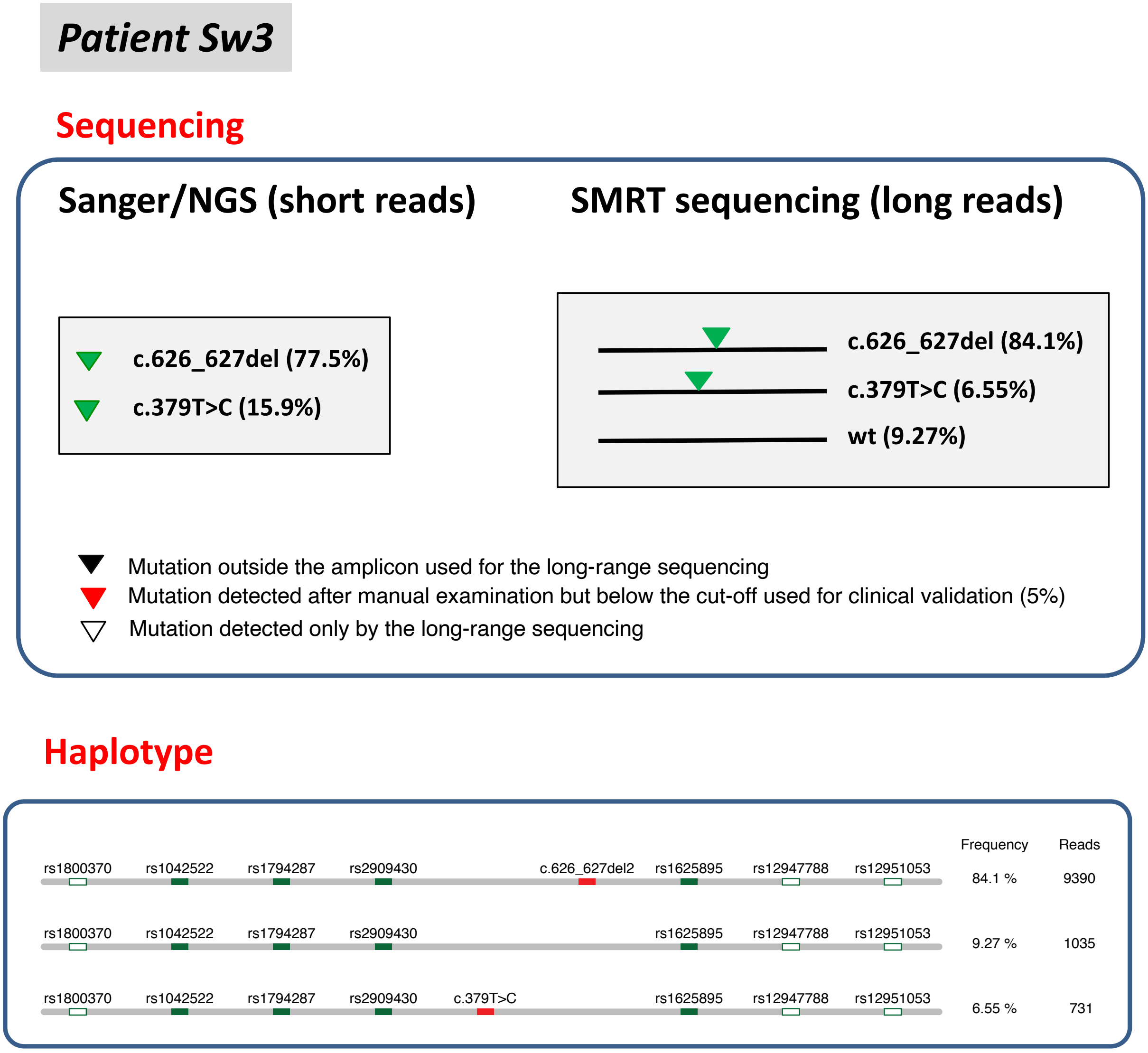

Supplementary Figure S11B

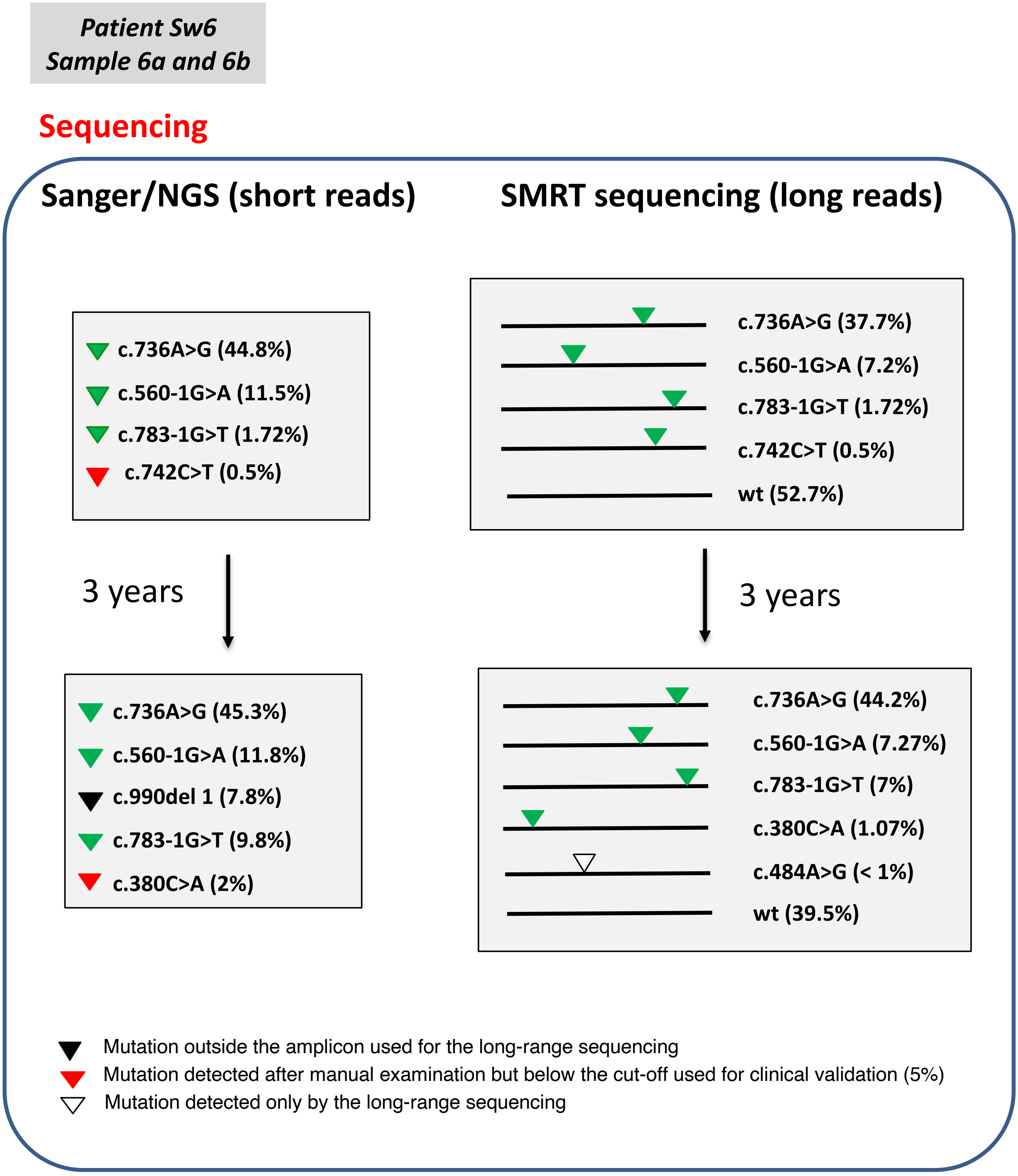

Supplementary Figure S11C

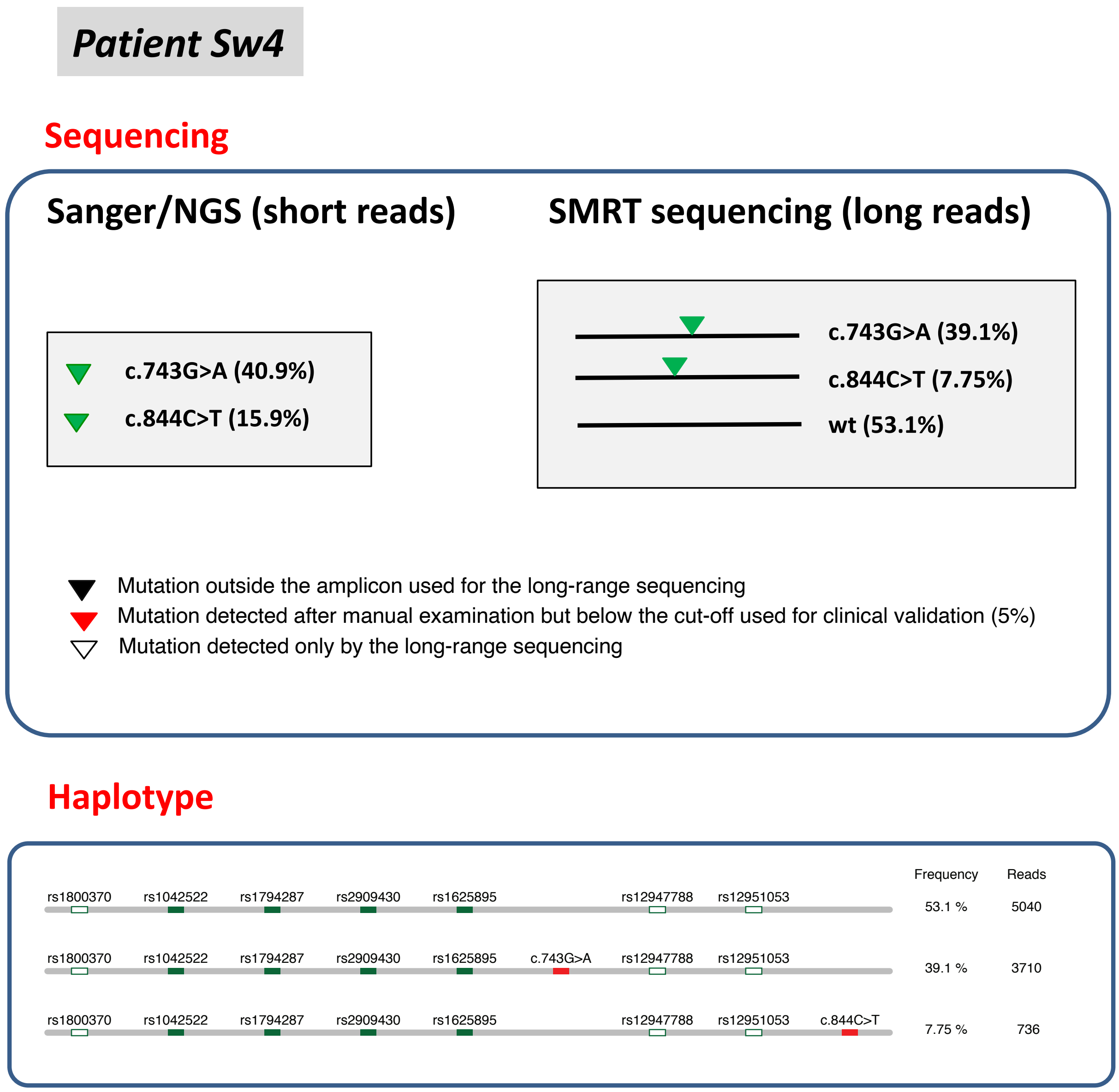

Supplementary Figure S11D

Patient Sw5

Sequencing

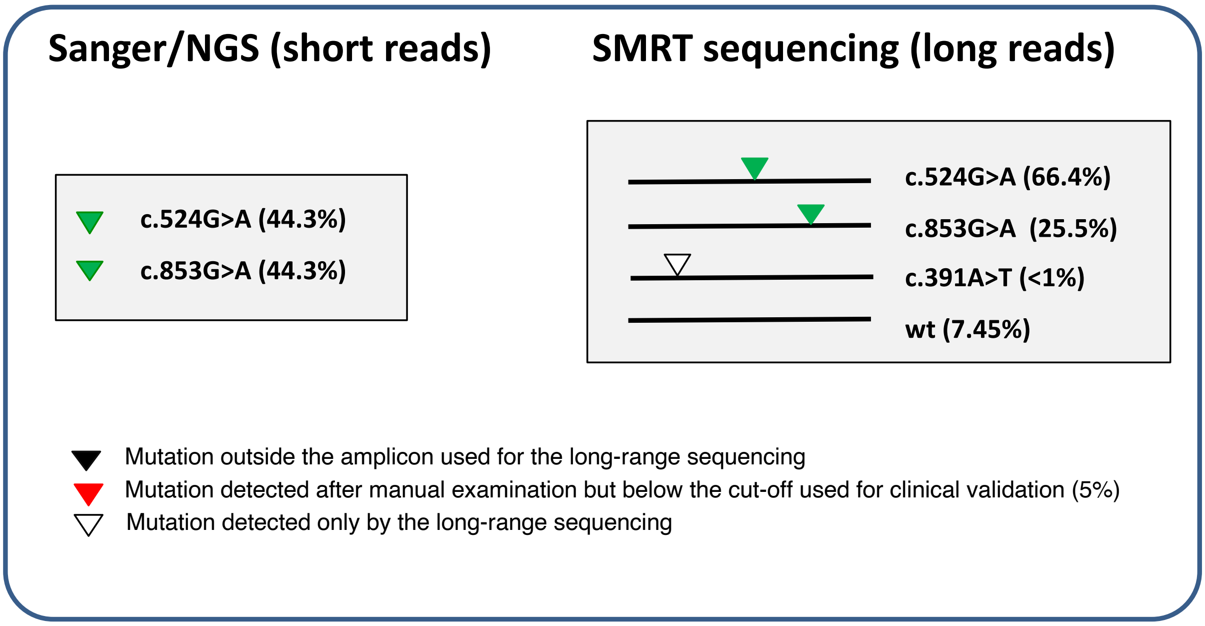

Haplotype

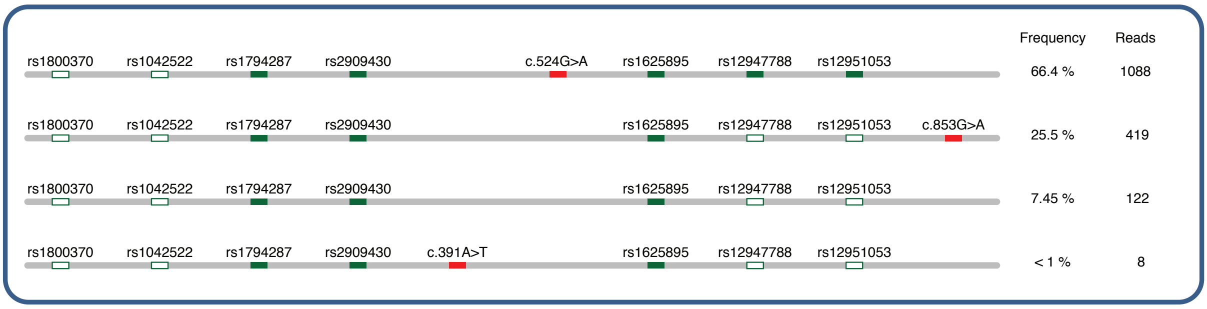

Supplementary Figure S11E

Supplementary figure S11 A to E: Long-range sequencing of CLL patients shows that *TP53* mutations in multi-mutated patients are in different alleles.

A to E: Detailed analysis of the 5 patients included in this study. For each patient, 2 sections are available i.e. sequencing and haplotype. Sequencing: conventional NGS analysis is shown on the left. No allelic distribution can be inferred from this type of analysis. SMRT sequencing is shown on the right. It provides an accurate picture of the allelic distribution of each *TP53* variant, as well as the remaining wt allele [6]. The frequencies of the different alleles are shown in parentheses. Haplotype: allelic distribution of all *TP53* variants (germline and somatic) according to the SMRT analysis. Somatic *TP53* variants are shown in red. Biallelic germline variants (SNPs) are shown in white (allele 1) and green (allele 2) to make a distinction for heterozygote cases.

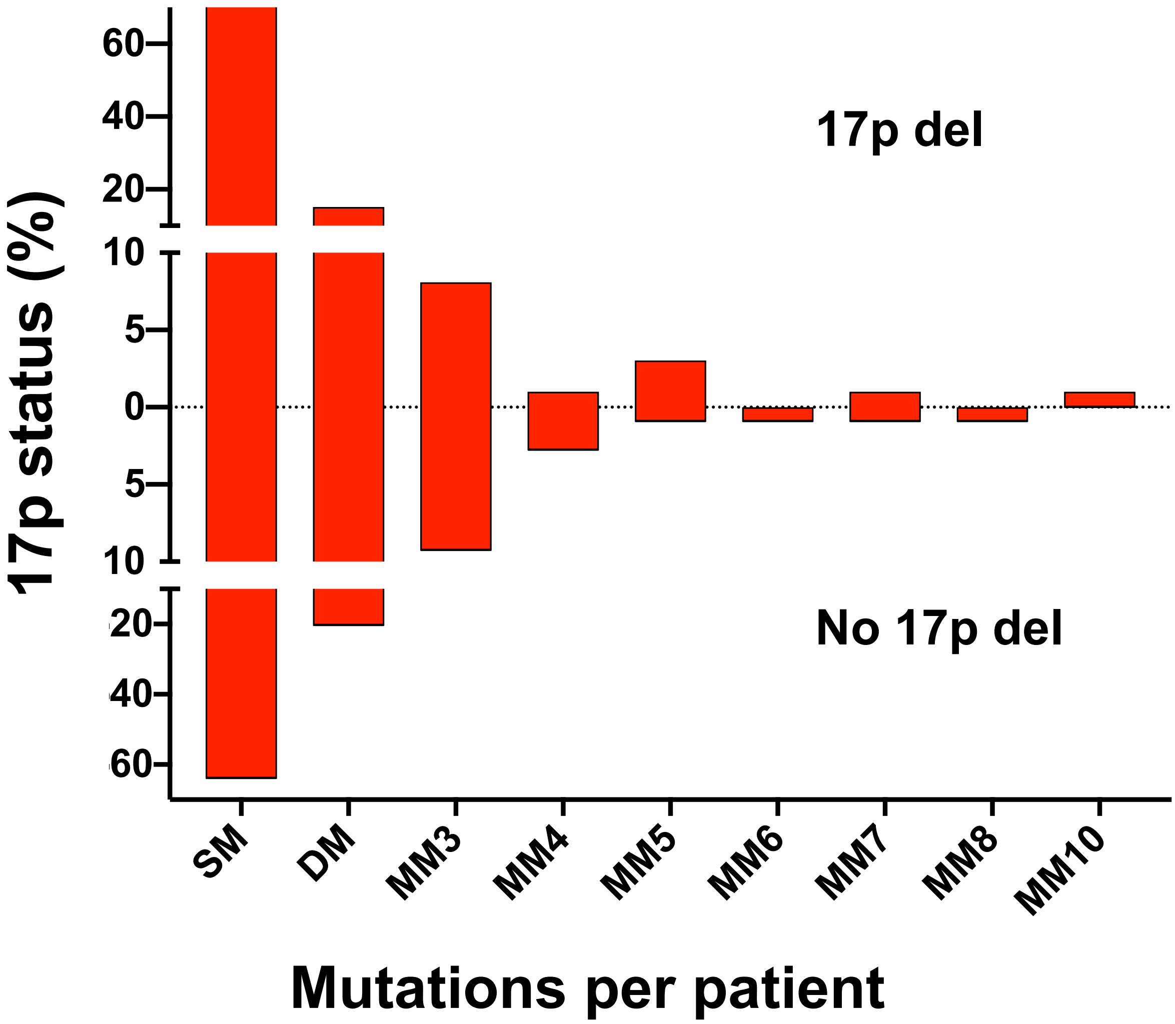

Supplementary Figure S12: Multiple *TP53* variants are found at the same frequency in patients with or without 17p deletion.

SM: patients with a single *TP53* mutation; DM: patients with two *TP53* mutations; MM3 to MM10: patients with three to ten *TP53* mutations.

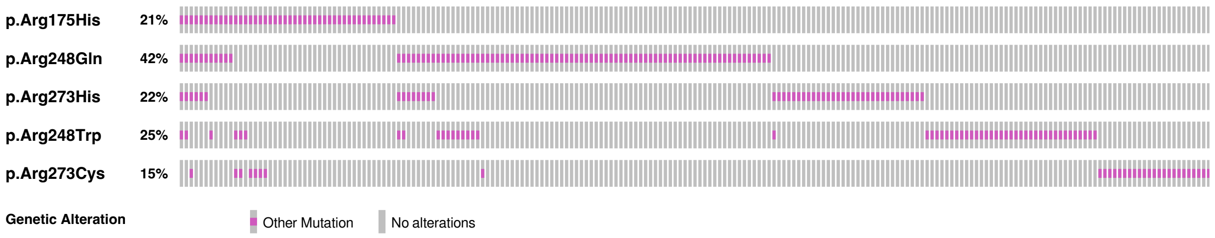

Supplementary Figure S13: Distribution of *TP53* hotspot variants in CLL patients.

CLL patients included in both UMD_CLL and the FILO cohort were used for this analysis. Data were analyzed by OncoPrinter (cBioPortal Version 1.14.0) [7].

Supplementary Table S1: Patient characteristics.

**Number of patients**	n=683
**Median age (years), [min;max]**	70 [23;98]
**Cytogenetics, n (%)**	
Trisomy 12	22/181 (12%)
Deletion 17p	127/257 (49%)
Deletion 11q	24/175 (14%)
Complex karyotype	62/169 (36%)
**IGHV status, n (%)**	
Mutated	204/464 (44%)
Unmutated<br><br>**Not previously treated**	260/464 (56%)<br><br>121/301 (40%)

Supplementary Table S2: Mutations summary.

	Number of variants (%)		
	Conventional sequencing	NGS	Total
Missense	136 (69.4)	632 (73.5)	768 (72.7)
Nonsense	17 (8.7)	52 (6)	69 (6.5)
Synonymous	1 (0.5)	0 (0)	1 (0.1)
Splice site	13 (6.6)	69 (8)	82 (7.8)
Intronic	4 (2)	0 (0)	4 (0.4)
Frameshift	19 (9.7)	93 (10.8)	112 (10.6)
Inframe	6 (3.1)	14 (1.6)	20 (1.9)
Total	196 (100)	860 (100)	1056 (100)
	Number of patients (%)		
Variants per patients	Conventional sequencing	NGS	Total
1 variant	150 (87.2)	351 (68.7)	501 (73.4)
2 variants	20 (11.6)	81 (15.9)	101 (14.8)
3 variants	2 (1.2)	41 (8)	43 (6.3)
4 and + variants	0 (0)	38 (7.4)	38 (5.6)
Total	172 (100)	511 (100)	683 (100)

Supplementary Table S3: Mutation description.

(Provided as an Excel file)

Table S4: Benign polymorphisms identified in CLL patients included in UMD.

HG19_Variant	Variant_Type	Event_type	Mutation_effect	Protein P1 TP53_alpha NP_000537.3	LRG_321t1	occurrence in UMD_CLL	ACMG_class
chr17:g.7577091G>A	SNV	G>A	Missense_variant	p.Arg283Cys	c.847C>T	16	Benign
chr17:g.7577069C>T	SNV	C>T	Missense_variant	p.Arg290His	c.869G>A	9	Benign
chr17:g.7577577T>C	SNV	T>C	Missense_variant	p.Asn235Ser	c.704A>G	8	Benign
chr17:g.7578464G>A	SNV	G>A	Missense_variant	p.Arg156Cys	c.466C>T	3	Benign
chr17:g.7579705C>T	SNV	C>T	Missense_variant	p.Val31Ile	c.91G>A	3	Benign
chr17:g.7579358C>T	SNV	C>T	Missense_variant	p.Arg110His	c.329G>A	2	Benign
chr17:g.7579470C>T	SNV	C>T	Missense_variant	p.Val73Met	c.217G>A	1	Benign
chr17:g.7579439G>A	SNV	G>A	Missense_variant	p.Ala83Val	c.248C>T	1	Benign

All these variants were defined as rare single nucleotide polymorphisms (SNPs) in the human population; they do not display any loss of function [8, 9]. These variants were removed from UMD_CLL for all analyses described in the present study.

References

1. Kundra R, Zhang H, Sheridan R, Sirintrapun SJ, Wang A, Ochoa A, Wilson M, Gross B, Sun Y, Madupuri R, Satravada BA, Reales D, Vakiani E, Al-Ahmadie HA, Dogan A, Arcila M, Zehir A, Maron S, Berger MF, Viaplana C, Janeway K, Ducar M, Sholl L, Dogan S, Bedard P, Surrey LF, Sanchez IH, Syed A, Rema AB, Chakravarty D, Suehnholz S, Nissan M, Iyer GV, Murali R, Bouvier N, Soslow RA, Hyman D, Younes A, Intlekofer A, Harding JJ, Carvajal RD, Sabbatini PJ, Abou-Alfa GK, Morris L, Janjigian YY, Gallagher MM, Soumerai TA, Mellinghoff IK, Hakimi AA, Fury M, Huse JT, Bagrodia A, Hameed M, Thomas S, Gardos S, Cerami E, Mazor T, Kumari P, Raman P, Shivdasani P, MacFarland S, Newman S, Waanders A, Gao J, Solit D, Schultz N OncoTree: A Cancer Classification System for Precision Oncology. JCO Clin Cancer Inform. 2021;5:221-230

2. Puente XS, Pinyol M, Quesada V, Conde L, Ordóñez GR, Villamor N, Escaramis G, Jares P, Beà S, González-Díaz M, Bassaganyas L, Baumann T, Juan M, López-Guerra M, Colomer D, Tubío JM, López C, Navarro A, Tornador C, Aymerich M, Rozman M, Hernández JM, Puente DA, Freije JM, Velasco G, Gutiérrez-Fernández A, Costa D, Carrió A, Guijarro S, Enjuanes A, Hernández L, Yagüe J, Nicolás P, Romeo-Casabona CM, Himmelbauer H, Castillo E, Dohm JC, de Sanjosé S, Piris MA, de Alava E, San Miguel J, Royo R, Gelpí JL, Torrents D, Orozco M, Pisano DG, Valencia A, Guigó R, Bayés M, Heath S, Gut M, Klatt P, Marshall J, Raine K, Stebbings LA, Futreal PA, Stratton MR, Campbell PJ, Gut I, López-Guillermo A, Estivill X, Montserrat E, López-Otín C, Campo E Whole-genome sequencing identifies recurrent mutations in chronic lymphocytic leukaemia. Nature. 2011;475:101-105

3. Lazarian G, Theves F, Hormi M, Letestu R, Eclache V, Bidet A, Cornillet-Lefebvre P, Davi F, Delabesse E, Estienne MH, Etancelin P, Kosmider O, Laibe S, Lode L, Muller M, Nadal N, Naguib D, Pastoret C, Poulain S, Sujobert P, Veronese L, Imache S, Lefebvre V, Cymbalista F, Baran-Marszak F, Soussi T, French Innovative Leukemia Organisation FILO TP53 mutations at codon 234 are associated with chlorambucil treatment in chronic lymphocytic leukemia. Am J Hematol. 2022;97:E159-E162

4. Bomben R, Rossi FM, Vit F, Bittolo T, D'Agaro T, Zucchetto A, Tissino E, Pozzo F, Vendramini E, Degan M, Zaina E, Cattarossi I, Varaschin P, Nanni P, Berton M, Braida A, Polesel J, Cohen JA, Santinelli E, Biagi A, Gentile M, Morabito F, Fronza G, Pozzato G, D'Arena G, Olivieri J, Bulian P, Pepper C, Hockaday A, Schuh A, Hillmen P, Rossi D, Chiarenza A, Zaja F, Di Raimondo F, Del Poeta G, Gattei V TP53 Mutations with Low Variant Allele Frequency Predict Short Survival in Chronic Lymphocytic Leukemia. Clin Cancer Res. 2021;27:5566-5575

5. Catherwood MA, Wren D, Chiecchio L, Cavalieri D, Donaldson D, Lawless S, ElHassadi E, Hayat A, Cahill MR, O'Shea D, Sargent J, Stewart P, Maurya M, Quinn J, Murphy P, de Castro DG, Mills K, Cross NCP, Forconi F, Iyengar S, Schuh A, Thornton P TP53 Mutations Identified Using NGS Comprise the Overwhelming Majority of TP53 Disruptions in CLL: Results From a Multicentre Study. Front Oncol. 2022;12:909615

6. Lodé L, Ameur A, Coste T, Ménard A, Richebourg S, Gaillard JB, Le Bris Y, Béné MC, Lavabre-Bertrand T, Soussi T Single-molecule DNA sequencing of acute myeloid leukemia and myelodysplastic syndromes with multiple TP53 alterations. Haematologica. 2018;103:e13-e16

7. Gao J, Aksoy BA, Dogrusoz U, Dresdner G, Gross B, Sumer SO, Sun Y, Jacobsen A, Sinha R, Larsson E, Cerami E, Sander C, Schultz N Integrative analysis of complex cancer genomics and clinical profiles using the cBioPortal. Sci Signal. 2013;6:pl1

8. Doffe F, Carbonnier V, Tissier M, Leroy B, Martins I, Mattsson JSM, Micke P, Pavlova S, Pospisilova S, Smardova J, Joerger AC, Wiman KG, Kroemer G, Soussi T Identification and functional characterization of new missense SNPs in the coding region of the TP53 gene. Cell Death Differ. 2021;28:1477-1492

9. Soussi T Benign SNPs in the Coding Region of TP53: Finding the Needles in a Haystack of Pathogenic Variants. Cancer Res. 2022;82:3420-3431
